# Reduced Bone Mass in Collagen Prolyl 4‐Hydroxylase *P4ha1* ^+/−^; *P4ha2* ^−/−^ Compound Mutant Mice

**DOI:** 10.1002/jbm4.10630

**Published:** 2022-05-09

**Authors:** Jussi‐Pekka Tolonen, Antti M Salo, Mikko Finnilä, Ellinoora Aro, Emma Karjalainen, Veli‐Pekka Ronkainen, Kati Drushinin, Christophe Merceron, Valerio Izzi, Ernestina Schipani, Johanna Myllyharju

**Affiliations:** ^1^ Oulu Center for Cell‐Matrix Research University of Oulu Oulu Finland; ^2^ Biocenter Oulu University of Oulu Oulu Finland; ^3^ Faculty of Biochemistry and Molecular Medicine University of Oulu Oulu Finland; ^4^ Research Unit of Medical Imaging, Physics and Technology, Faculty of Medicine University of Oulu Oulu Finland; ^5^ Departments of Orthopaedic Surgery, Medicine, and Cell and Developmental Biology University of Michigan School of Medicine Ann Arbor MI USA; ^6^ Research Unit of Biomedicine, Faculty of Medicine University of Oulu Oulu Finland; ^7^ Finnish Cancer Institute Helsinki Finland; ^8^ Present address: McKay Laboratory, Department of Orthopedic Surgery University of Pennsylvania‐Perelman Medical School Philadelphia PA USA

**Keywords:** BONE HISTOMORPHOMETRY, BONE μCT, COLLAGEN, GENETIC ANIMAL MODELS, OSTEOBLASTS

## Abstract

Proper deposition of the extracellular matrix and its major components, the collagens, is essential for endochondral ossification and bone mass accrual. Collagen prolyl 4‐hydroxylases (C‐P4Hs) hydroxylate proline residues in the ‐X‐Pro‐Gly‐ repeats of all known collagen types. Their product, 4‐hydroxyproline, is essential for correct folding and thermal stability of the triple‐helical collagen molecules in physiological body temperatures. We have previously shown that inactivation of the mouse *P4ha1* gene, which codes for the catalytic α subunit of the major C‐P4H isoform, is embryonic lethal, whereas inactivation of the *P4ha2* gene produced only a minor phenotype. Instead, mice with a haploinsufficiency of the *P4ha1* gene combined with a homozygous deletion of the *P4ha2* gene present with a moderate chondrodysplasia due to transient cell death of the growth plate chondrocytes. Here, to further characterize the bone phenotype of the *P4ha1* ^+/−^; *P4ha2* ^−/−^ mice, we have carried out gene expression analyses at whole‐tissue and single‐cell levels, biochemical analyses, microcomputed tomography, histomorphometric analyses, and second harmonic generation microscopy to show that C‐P4H α subunit expression peaks early and that the C‐P4H deficiency leads to reduced collagen amount, a reduced rate of bone formation, and a loss of trabecular and cortical bone volume in the long bones. The total osteoblast number in the proximal *P4ha1* ^+/−^; *P4ha2* ^−/−^ tibia and the C‐P4H activity in primary *P4ha1* ^+/−^; *P4ha2* ^−/−^ osteoblasts were reduced, whereas the population of osteoprogenitor colony‐forming unit fibroblasts was increased in the *P4ha1* ^+/−^; *P4ha2* ^−/−^ marrow. Thus, the *P4ha1* ^+/−^; *P4ha2* ^−/−^ mouse model recapitulates key aspects of a recently recognized congenital connective tissue disorder with short stature and bone dysplasia caused by biallelic variants of the human *P4HA1* gene. Altogether, the data demonstrate the allele dose‐dependent importance of the C‐P4Hs to the developing organism and a threshold effect of C‐P4H activity in the proper production of bone matrix. © 2022 The Authors. *JBMR Plus* published by Wiley Periodicals LLC on behalf of American Society for Bone and Mineral Research.

## 1 Introduction

Collagen prolyl 4‐hydroxylases (C‐P4Hs, EC 1.14.11.2) catalyze the formation of 4‐hydroxyproline (4Hyp) in the procollagen α chains that trimerize to form functional triple‐helical collagen molecules.^(^ ^1^, ^2^, ^3^ ^)^ The simple hydroxylation ensures the appropriate folding and thermal stability of the triple‐helical collagen molecules at physiological human body temperature.^(^ ^2^, ^4^, ^5^, ^6^ ^)^ Because collagens comprise a third of the total protein mass in humans, the formation of 4Hyp represents one of the most common posttranslational modifications of mammal proteins.^(^ ^5^ ^)^

The C‐P4Hs are 2‐oxoglutarate‐dependent dioxygenases (2‐OGDDs) with a α_2_β_2_ tetramer composition.^(^ ^2^, ^7^, ^8^ ^)^ Like all 2‐OGDDs, they require 2‐oxoglutarate, Fe^2+^, molecular oxygen, and vitamin C as a reducing agent to hydroxylate proline residues in the ‐X‐Pro‐Gly‐ repeats of 28 known collagen types and more than 20 proteins with collagen‐like domains such as the complement C1q protein.^(^ ^3^, ^9^, ^10^ ^)^ Because the C‐P4H reaction is dependent on 2‐oxoglutarate (a Krebs cycle metabolite), vitamin C, and molecular oxygen, the C‐P4Hs participate in a vast network of metabolic sensors and regulators of tissue homeostasis^(^ ^11^, ^12^, ^13^ ^)^ and are also involved in cancer and fibrotic disease.^(^ ^14^, ^15^, ^16^, ^17^ ^)^ As such, small‐molecule inhibitors of the C‐P4Hs and other closely related 2‐OGDDs (ie, hypoxia‐inducible factor [HIF]‐P4Hs known as PHDs) are targets of active research.^(^ ^18^, ^19^ ^)^

The α subunits of the C‐P4H holoenzyme contain the substrate binding domains and catalytic sites, whereas the β subunits that are identical to protein disulphide isomerase prevent α subunit aggregation.^(^ ^20^, ^21^, ^22^, ^23^, ^24^ ^)^ Three human genes, namely *P4HA1*, *P4HA2*, and *P4HA3*, code for the α subunit isoforms, forming C‐P4H‐I ([α(I)_2_β_2_]), C‐P4H‐II ([α(II)_2_β_2_]), and C‐P4H‐III ([α(III)_2_β_2_]) isoenzymes.^(^ ^25^, ^26^, ^27^ ^)^ The main C‐P4H isoform in most cells and tissues is C‐P4H‐I and a total knockout of *P4ha1* is embryonic lethal in mice after E10.5 due to disruption of the basement membranes.^(^ ^28^ ^)^ Furthermore, because a complete *P4ha2* knockout results in only a minor phenotype, C‐P4H‐I is most likely capable of largely compensating for a loss in C‐P4H‐II activity.^(^ ^29^ ^)^ Very little is currently known about C‐P4H‐III, the α(III) subunit mRNA being expressed at low levels in the fibrous cap of atherosclerotic plaques and in a number of fetal and adult tissues, and cancer.^(^ ^17^, ^25^, ^30^ ^)^

Our understanding of the roles of C‐P4H activity and individual isoenzymes in human development and tissue homeostasis gained a significant step forward with the recent discovery of the first congenital connective tissue disorder caused by elaborate biallelic pathogenic variants in *P4HA1*.^(^ ^31^ ^)^ These variants resulted in a 50% decrease of the total C‐P4H activity, presenting with a short stature, bone dysplasia, joint contractures, muscle weakness, and motor function impairment, which improved over time. On the other hand, pathogenic variants in *P4HA2* are associated with myopia.^(^ ^32^ ^)^ However, the tissue‐specific roles of C‐P4H activity and isoenzymes still remain unclear. Because complete inactivation of *P4ha1* is embryonic lethal, and heterozygous as well as homozygous knockouts of *P4ha1* and *P4ha2*, respectively, do not result in obvious anatomical or histological impairments, we generated a compound mutant mouse line with a haploinsufficiency of *P4ha1* and a homozygous deletion of *P4ha2*.^(^ ^29^ ^)^ These *P4ha1* ^+/−^; *P4ha2* ^−/−^ mice are smaller than their wild‐type counterparts and present with transient chondrocyte cell death in the growth plate, resulting in chondrodysplasia.^(^ ^29^ ^)^

In the present study, we investigate the bone phenotype of the *P4ha1* ^+/−^; *P4ha2* ^−/−^ mice, which recapitulates central aspects of the human connective tissue disorder caused by biallelic *P4HA1* mutations,^(^ ^31^ ^)^ to explore the role of collagen prolyl 4‐hydroxylation in the collagen‐rich bone matrix.

## 2 Materials and Methods

### 2.1 Gene‐modified mouse lines

The generation of the C57BL/6JOlaHsd knockout mouse lines for *P4ha1* and *P4ha2* has been described previously.^(^ ^28^, ^29^ ^)^ The mice were backcrossed at least 10 times. *P4ha1* ^+/−^ and *P4ha2* ^−/−^ were crossbred to generate *P4ha1* ^+/−^; *P4ha2* ^+/−^ mice, which were then further intercrossed with *P4ha2* ^−/−^. The genotypes obtained from these crosses were *P4ha2* ^+/−^, *P4ha1* ^+/−^; *P4ha2* ^+/−^, *P4ha2* ^−/−^, and *P4ha1* ^+/−^; *P4ha2* ^−/−^. This crossbreeding strategy was chosen according to the 3R principle of animal experiments to reduce the number of mice needed to obtain sufficient numbers of the *P4ha1* ^+/−^; *P4ha2* ^−/−^ genotype for the planned experiments. Because this crossbreeding strategy did not produce any wild‐type littermates, the *P4ha2* ^+/−^ mice were used as controls. Also, as live *P4ha1* ^+/−^; *P4ha2* ^−/−^ pups are born in a sub‐Mendelian ratio,^(^ ^29^ ^)^ to keep the size of the mouse colony required for the present study at a reasonable scale, we selected to use mainly female mice in the experiments. The number of mice used in each analysis is given in the figure legends. The C57BL6 background line is known to be relatively osteopenic.^(^ ^33^ ^)^

The primers used for genotyping are listed in Table 1.^(^ ^29^ ^)^ The genotypes of the offspring were verified by PCR with a forward primer from intron 1 of the *P4ha1* gene and a reverse primer from either exon 2 or the *LacZ* gene, which is present in the knockout targeting construct. The PCR products are either 1.5 kb or 850 bp in size, respectively. The primers for the *P4ha2* gene included a forward primer from intron 2 and a reverse primer either from intron 3 or the *LacZ* gene, with PCR products of 1.9 and 2.0 kb in size, respectively.

**Table 1:** Sequences for PCR Primers Used in Mouse Genotyping

Gene	Name	Sequence (5′–3′)
*P4ha1*	P4ha1 intron 1 forward	GCATAGAACACAGAAGTAAGAGAAA
	P4ha1 exon 2 reverse	GCATAGAACACAGAAGTAAGAGAAA
*P4ha2*	P4ha2 intron 2 forward	TGAGCCATTCCGAGATTTGGTTTA
	P4ha2 exon 3 reverse	AGGGCATTGGTTTTCTAAGGGCGC
*LacZ*	LacZ rev	ACCCTGCCATAAAGAAACTGT

Animal maintenance and experiments were approved by the Animal Care and Use Committee of the University of Oulu and the National Animal Experiment Board of Finland, license numbers ESAVI/5307, ESAVI/259, and ESAVI/8179. During the study, the animals were observed daily.

### 2.2 Sample preparation and histomorphometrical analyses

Six‐week‐ and 3‐month‐old female mice were used for the histomorphometric and microcomputed tomography (μCT) analyses. Tibias and femurs were harvested in phosphate‐buffered saline (PBS) on ice after euthanization, dissected free from soft tissue, and fixed in fresh 10% neutral‐buffered formalin at 4°C for 1 day, after which they were stored in 70% ethanol until further procedures.

In experiments where the rate of extracellular matrix (ECM) mineralization was analyzed by fluorescence microscopy, the mice were injected i.p. with 40 mg/kg calcein (dissolved in 0.9% NaCl, 0.2% NaHCO_3_), a fluorophore that binds to mineralizing ECM, at 6 and 2 days before death.

Histomorphometric analyses were performed on the proximal tibias. The right tibias were decalcified in 10% EDTA for 2 weeks and the decalcified samples were processed and embedded in paraffin according to standard procedures. The decalcified tibias were cut in full into 5‐μm paraffin slides that were used for hematoxylin and eosin (H&E) staining and tartrate‐resistant acid phosphatase (TRAP) staining. Every fifth paraffin section was stained with H&E to locate the midsection of the metaphysis to carry out the TRAP staining, which was then performed according to manufacturer's instructions (Sigma‐Aldrich, St. Louis, MO, USA; 387A‐1KT). The undecalcified left tibias were embedded in poly(methyl methacrylate) plastic. The plastic embedded samples were cut into 5‐ and 8‐μm‐thick consecutive sections with the Polycut microtome (Reichert‐Jung, Leica, Wetzlar, Germany). The 5‐μm plastic slides were stained using Masson‐Goldner's trichrome. The 8‐μm plastic slides were coverslipped without staining.

An Olympus (Tokyo, Japan) BX51 microscope was used to visualize the 5‐μm paraffin and plastic‐embedded slides and the fluorescent 8‐μm slides at 20× magnification. The images were stitched using the Microsoft Image Composite Editor. For the analyses of total tissue volume (TV), trabecular bone volume (BV), and osteoblast (N.Ob) and osteoclast (N.Oc) numbers, the metaphyseal region of interest (ROI) was defined as an area 500 μm in height below the growth plate. The growth plate, cortical bone, and cortical osteoblasts were excluded from the analyses. The histomorphometric analyses follow the guidelines provided by the American Society for Bone and Mineral Research Histomorphometry Nomenclature Committee.^(^ ^34^ ^)^

### 2.3 Microcomputed tomography (μCT)

Tibias and femurs were collected and fixed in formalin as described above and scanned using Skyscan 1176 μCT with high‐resolution settings (9‐μm isotropic voxel size). Femoral and tibial lengths were determined in Dataviewer, and the cortical and trabecular bone morphologies were calculated from reconstructed 3D images using CTAn software (Skyscan, Kontich, Belgium), selecting ROIs from anatomically matched locations. Although the shortening of the long bones in the *P4ha1* ^+/−^; *P4ha2* ^−/−^ mice is statistically significant,^(^ ^29^ ^)^ the difference is so small that it would only have minimal (if any) effects on the histomorphometric analyses and the regions of interest were therefore not scaled to the length of the bone. Calcium hydroxyapatite phantoms were used to calibrate bone mineral density. The μCT data have been reported in accordance with the guidelines provided by the American Society for Bone and Mineral Research.^(^ ^35^ ^)^

### 2.4 Amino acid analysis and second harmonic generation (SHG) microscopy

Amino acid analysis of tibial bone was performed as described previously.^(^ ^29^ ^)^ To explore qualitative changes in the structure of the bone ECM, SHG microscopy was used to analyze the composition and alignment of collagen fibrils of control and *P4ha1* ^+/−^; *P4ha2* ^−/−^ bone ECM at 3 months of age.^(^ ^36^, ^37^ ^)^ A more detailed description of the materials and methods used in SHG analysis is given in Supplemental Materials and Methods.

### 2.5 Mechanical testing by three‐point bending

Mechanical testing was performed on the long bones of 6‐week‐old male mice at three sites: the femoral midshaft, the femoral neck, and the tibial midshaft. The bones were dissected free from soft tissue and stored in PBS at −20°C. Bone strength was measured on an Instron 3366 Universal Tabletop Testing System (Instron Corp., Norwood, MA, USA).^(^ ^38^, ^39^, ^40^ ^)^ Briefly, both tibias and femurs were tested in three‐point bending by placing the bones on the support with the anterior surface facing upward. The span length was set at 7.5 mm. To test the femoral neck, the proximal half of the femur was placed on a lab‐made holder and the femoral neck was loaded axially until fracture. For all tests, the load was applied with a constant speed of 0.155 m/s. The average of the left and right bones was used as the final result.

### 2.6 Colony‐forming unit‐fibroblast (CFU‐F) isolation and in vitro matrix production assays

Female mice were euthanized at 5 weeks of age to harvest both hind legs. After the removal of the skin, the hind legs were kept in Hank's Balanced Salt Solution (HBSS, Gibco, Thermo Fisher Scientific, Waltham, MA, USA) on ice. To harvest the tibias and femurs, the hind legs were briefly dipped in 70% ethanol and put in PBS, where the remaining muscle and connective tissues were removed. To isolate the bone marrow, the tibias were cut at the insertion of the patella tendon and distal tibiofibular joint, and the femurs were cut slightly distally to the capsule attachment and under the minor trochanter. The bone marrow was flushed with 10 mL HBSS per bone through a 40‐μm cell strainer (Falcon) into a 50‐mL Falcon tube. The isolated cells were centrifuged at 240*g*   for 5 minutes, and then resuspended in minimum essential medium α (MEM α, GlutaMAX, Gibco) supplemented with 20% fetal bovine serum (Biowest, Riverside, MO, USA), 1% penicillin–streptomycin (Sigma‐Aldrich), and 0.1% amphotericin B (Gibco). An amount of 16 × 10^6^ cells per well were plated on a 6‐well plate (Corning Primaria, Corning Inc., Corning, NY, USA; 353846) and incubated for 24 hours in 5% CO_2_ at 37°C. The cells were washed several times at days 1, 4, and 7 with HBSS to remove free‐floating hematopoietic cells. Finally, the cells were stained with an Alkaline Phosphatase Kit (Sigma‐Aldrich, 86C‐1KT) and counterstained with Neutral Red solution included in the kit, according to the manufacturer's instructions, to identify mesenchymal stromal cell (ie, CFU‐Fs) colonies at day 10. Colonies of 10 positive cells were included in the analyses.

To stimulate matrix production by the CFU‐F colonies, the cells were isolated as described above and incubated in an osteogenic medium: MEM α (GlutaMAX, Gibco) supplemented with 20% fetal bovine serum, 1% penicillin–streptomycin, 0.1% amphotericin B, 50 μg/mL l‐ascorbic acid phosphate (Wako, Richmond, VA, USA), and 10 mM glycero‐2‐phosphate disodium salt pentahydrate (Sigma). The cells were washed several times at days 1, 4, and 7 with HBSS, after which the medium was changed every 3 days. Finally, the cells were stained with Alizarin Red S (ARS) at day 28. The amount of ARS was quantified by the absorption method.^(^ ^41^ ^)^ All in vitro studies were performed at pO_2_ 21%, a standard but non‐physiological condition for cell culture studies.

### 2.7 Measurement of total collagen prolyl 4‐hydroxylase activity

Female mice were euthanized at 5 weeks of age to harvest both tibias and femurs as described for the isolation of the CFU‐F cells. After removing the bone marrow, the bones were diced into smaller pieces (approximately 2 × 2 mm) and digested with 2 mg/mL collagenase (Worthington, Lakewood, NJ, USA; collagenase type 2) in 5% fetal bovine serum (Biowest)‐MEM α for 1 hour at 37°C. The bone chips were cultured on a 10‐cm cell culture plate in 10% fetal bovine serum (Biowest)‐MEM α (including 1% penicillin–streptomycin, 0.1% amphotericin B) to allow the migration of osteoblasts. The medium was changed every 3 days. The bone chips were allowed to settle on the cell culture plate until a sufficient coverage of osteoblasts was observed after which the bone chips were removed, and the cells were harvested at day 28.

The osteoblasts were lysed on ice in 137 mM NaCl, 20 mM Tris‐HCl, pH 8, 10% glycerol, 1% Nonidet P‐40, and cOmplete proteinase inhibitor without EDTA (Roche, Mannheim, Germany). The protein concentration was determined using the Bio‐Rad protein assay (Bio‐Rad, Hercules, CA, USA). The total C‐P4H activity was measured by the formation of 4‐hydroxy [^14^C]proline in [^14^C]proline‐labeled non‐hydroxylated pro‐collagen α chains of chick type I collagen.^(^ ^42^ ^)^

### 2.8 Measurement of serum markers by enzyme immunoassays

Female mice were harvested at 5 weeks of age. Blood samples were collected immediately after euthanization by opening the peritoneal cavity and revealing the posterior vena cava for puncture using a 25G needle and a 1‐mL syringe. The blood samples were collected into Micro 1.1‐mL Z‐Gel tubes (Sarstedt, Numbrecht, Germany) and centrifuged at 10,000*g* for 10 minutes at room temperature to separate the serum. Serum levels for the N‐terminal propeptide of type I procollagen (PINP) and cross‐linked C‐terminal telopeptide of type I collagen (CTX‐I) were measured using commercial enzyme immunoassay (EIA) kits (Rat/Mouse PINP EIA and RatLaps [CTX‐I] EIA, respectively) (Immunodiagnostic Systems, Boldon, UK), according to the manufacturer's instructions. The mice were not fasted before CTX‐I measurements.

### 2.9 Digital droplet polymerase chain reaction (ddPCR) analysis

Wild‐type C57BL6/N male mice were euthanized at the following time points: newborn (P0), day 2 (P2), day 4 (P4), 1, 2, and 6 weeks. The following tissues were harvested: femur, tibia, bone marrow (6 weeks, obtained by flushing the tibia), growth plate of proximal end of tibia, proximal epiphysis of tibia (6 weeks), and distal epiphysis of femur (6 weeks). Six‐week‐old bones were flushed clear of bone marrow, whereas younger bone samples contained both the diaphyses and bone marrow. Total RNA was isolated using either E.Z.N.A Total RNA Kit I (Omega Bio‐Tek, Norcross, GA, USA) or Trizol (Thermo Fisher Scientific) as per manufacturer's instructions. The iScript cDNA synthesis kit (Bio‐Rad) was used for reverse transcription according to the manufacturer's instructions.

A QX200 Droplet Digital PCR system (Bio‐Rad) was used for detection of *P4ha1*, *P4ha2*, and *P4ha3* expression. For each reaction, 2× ddPCR Supermix for probes (Bio‐Rad) was mixed with 250 nM TaqMan Gene Expression Assay (FAM) components (Thermo Fisher Scientific) and cDNA template (33 ng) to a final volume of 22 μL. The TaqMan Gene Expression Assays (FAM) probes used were P4ha1 (Mm00803137_m1), P4ha2 (Mm00477940_m1), and P4ha3 (Mm00622868_m1). From the samples, 20 μL were loaded onto an eight‐channel cartridge (Bio‐Rad) along with 70 μL of droplet generation oil for probes (Bio‐Rad). After emulsion generation on the QX200 Droplet Generator (Bio‐Rad), samples were transferred to a 96‐well PCR plate, heat‐sealed with foil by using PX1 PCR plate Sealer (Bio‐Rad), and amplified in T100 Thermal Cycler (Bio‐Rad). Thermal cycling conditions were 95°C for 10 minutes, followed by 40 cycles of 94°C for 30 seconds, 60°C for 1 minute, and 98°C for 10 minutes with a ramp rate of 2°C/s. Droplets were analyzed with QX 200 Droplet Reader (Bio‐Rad). Results were determined using the QuantaSoft software (Bio‐Rad) to calculate relative expression and copies/ng of RNA input.

### 2.10 Analysis of single‐cell RNAseq data

The data used in this study are sourced from GSE108892.^(^ ^43^ ^)^ Raw data were imported and reanalyzed using Seurat^(^ ^44^ ^)^ and other necessary packages in the R toolkit. All analyses were performed as in the original publication.

### 2.11 Statistical methods

The histomorphometrical analyses were carried out with Bioquant OSTEO (Bioquant, Nashville, TN, USA) in a single‐blinded fashion. Data analyses were performed using GraphPad Prism (software version 8, GraphPad, LaJolla, CA, USA). The Shapiro–Wilk test was used to assess the normality of each group. Student's *t* test was performed to compare two groups of data. One‐way ANOVA followed by post hoc Dunnett's multiple comparisons test was used to perform the statistical analyses of three or more groups against the control *P4ha2* ^+/−^ mice. A *p* value <0.05 was considered statistically significant. The box and whisker plots show all individual data points, statistically significant *p* values, median, and interquartile range (25th to 75th percentile). Sample size requirements were estimated from previous experiments.

## 3 Results

### 3.1 Expression of the C‐P4H α subunits in bone

The short stature and bone dysplasia associated with the biallelic pathogenic variants of the *P4HA1* gene imply that C‐P4Hs are involved in endochondral ossification and bone mass accrual.^(^ ^31^ ^)^ We have previously shown that a deficiency of collagen prolyl 4‐hydroxylation through a combined haploinsufficiency of *P4ha1* and homozygous deletion of *P4ha2* in a *P4ha1* ^+/−^; *P4ha2* ^−/−^ mouse line causes moderate chondrodysplasia.^(^ ^29^ ^)^ Furthermore, others have shown that increased posttranslational modifications of collagen, including prolyl 4‐hydroxylation, in the chondrocyte increases bone mass because the overmodified collagen molecules in the cartilaginous matrix resist protease‐mediated degradation.^(^ ^45^ ^)^ To expand on the significance of C‐P4H activity in the assembly of bone matrix, we first set out to quantify the relative expression levels and copies per ng of input RNA of the *P4ha1*, *P4ha2*, and *P4ha3* genes by ddPCR at different time points.

**Fig. 1:**
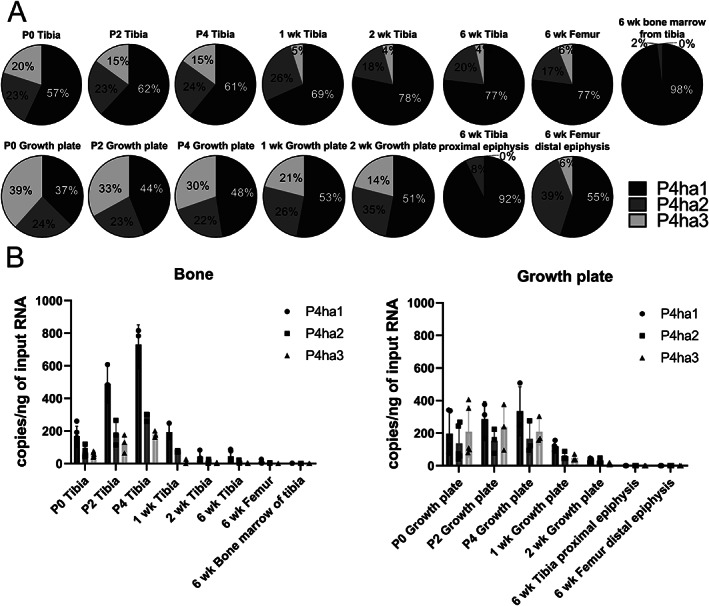
Digital droplet PCR (ddPCR) analysis of *P4ha1*, *P4ha2*, and *P4ha3* expression in the murine tibia, femur, and the growth plate of the tibia. ddPCR was used to study (*A*) the relative expression levels of *P4ha1*, *P4ha2*, and *P4ha3* and (*B*) the expression levels of *P4ha1*, *P4ha2*, and *P4ha3* in copies/ng of input RNA. Tibias and growth plates were harvested from newborn (P0), 2‐day‐old (P2), 4‐day‐old (P4), 1‐week‐old (1 week), 2‐week‐old (2 weeks), and 6‐week‐old (6 weeks) male mice, whereas femur and bone marrow from the tibia were harvested only from 6‐week‐old male mice (*n* = 3–5). The data in *B* are shown as bar plots with all individual data points.

In line with previous studies,^(^ ^28^ ^)^ *P4ha1* mRNA was in general the most abundant isoform, whereas the expression of *P4ha2* and *P4ha3* was lower and more variable between time points and anatomical locations (Fig. 1*A*, *B*). Interestingly, *P4ha3* expression surpassed that of *P4ha2* in the growth plate at early postnatal time points from P0 to P4 and was even higher than *P4ha1* expression at one time point, namely, P0 in the growth plate (Fig. 1*A*, *B*). The relative *P4ha1* transcript abundance increased with age, whereas those of *P4ha2* and *P4ha3* appeared to decrease (Fig. 1*A*). Absolute expression levels were highest at the early time points, peaking at P4, and then decreasing substantially by the 2‐week time point (Fig. 1*B*). Finally, the C‐P4H α subunit expression pattern in the femur was comparable to that of the tibia at 6 weeks, whereas the bone marrow expressed almost solely *P4ha1* at 6 weeks.

We next analyzed the mRNA expression of the C‐P4H α subunits, as well as other collagen hydroxylases and collagen types, using an RNAseq data set of adult mouse nonhematopoietic niches of the bone marrow at singe‐cell resolution.^(^ ^43^ ^)^ Expression of the C‐P4H α subunits, prolyl 3‐hydroxylases (*P3h1*, *P3h2*, and *P3h3*), and lysyl hydroxylases (*Plod1*, *Plod2* and *Plod3*) varied across the different stromal cell types, ranging from ubiquitous (*P4ha1* and *Plod3*) to cell‐specific, such as *P4ha3* (almost only in osteoblasts [OBs]), *Plod2* (vascular cells), and *P3h3* (perivascular cells and OBs; Supplemental Fig. S1 *A–C*). Among the OB populations profiled, the three clusters mark the process of differentiation from osteogenic precursors (O2) to mature OB (O3), also spanning the myeloid‐supportive population of O1^(^ ^43^ ^)^ that resembles closely the myofibroblasts of myeloid origin observed recently^(^ ^46^ ^)^ (Supplemental Fig. S1 *B, C*). Notably, expression of the C‐P4H α subunits and other collagen hydroxylases varied considerably along this trajectory, with *P4ha1* and *P4ha2* counts increasing in tune with OB commitment, whereas *P4ha3* marks almost exclusively the O1 population (Supplemental Fig. S1 *C*). Collagen expression within the same cells follows the expected distribution, with type I (*Col1a1* and *Col1a*2) and type II (*Col2a1*) collagen being almost exclusively expressed in mature OBs (O3) and the basal lamina marker type IV (*Col4a1*, *Col4a2*, and *Col4a3*) collagen characterizing vascular cells (V1) (Supplemental Fig. S1 *B*). In line with the differential expression of the C‐P4H α subunits, collagen expression changes across OB subpopulations, with the *P4ha3* ^High^ O1 cells shifting toward types III, V, and VI, all previously reported to play significant roles in osteo‐, fibro‐, and myogenesis,^(^ ^47^, ^48^, ^49^ ^)^ further suggesting that O1 and its collagen/collagen‐modifying machinery setup demarks a multifunctional population endowed with both hematopoietic and osteogenic support roles.

### 3.2 A significant loss of bone mass in the *P4ha1* ^+/−^; *P4ha2* ^−/−^ long bones

**Fig. 2:**
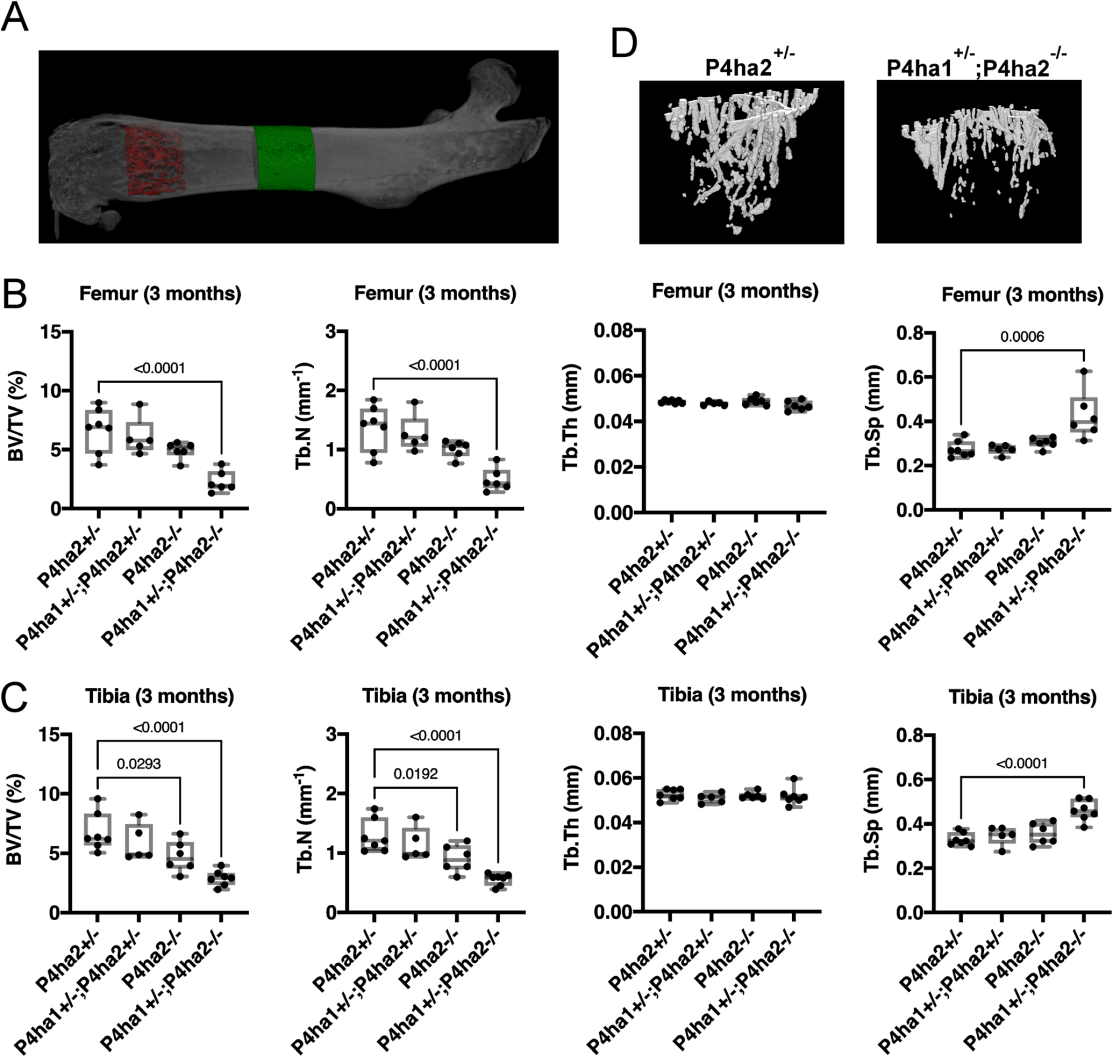
Decreased trabecular bone volume in the femur and tibias of 3‐month‐old *P4ha1* ^+/−^; *P4ha2* ^−/−^ mice. (*A*) A representative μCT‐generated image of the femur showing the regions of interest used for trabecular (red) and cortical (green) bone measurements. (*B*, *C*) Quantification of bone volume/tissue volume (BV/TV), trabecular number (Tb.N), trabecular thickness (Tb.Th), and trabecular separation (Tb.Sp) in the distal femur (*B*) and the proximal tibia (*C*) at 3 months of age. (*D*) Representative μCT‐generated images of the trabeculae in the proximal tibia of control *P4ha2* ^+/−^ (left) and *P4ha1* ^+/−^; *P4ha2* ^−/−^ (right) mice. The data in *B* and *C* are shown as box and whisker plots including all individual data points, median, and interquartile range (25th to 75th percentile). Statistical analysis was done with one‐way ANOVA followed by post hoc Dunnett's multiple comparisons test against the control *P4ha2* ^+/−^ mice, *n* = 5–7 mice/genotype. Statistically significant *p* values are shown in the graphs.

Next, to explore the impact of the C‐P4H deficiency in the *P4ha1* ^+/−^; *P4ha2* ^−/−^ mice, we measured the trabecular and cortical bone volumes in the long bones of female *P4ha1* ^+/−^; *P4ha2* ^−/−^ mice by μCT at two time points (Fig. 2*A*). At 6 weeks of age, trabecular bone volume fraction (BV/TV) follows a decreasing trend in the distal *P4ha1* ^+/−^; *P4ha2* ^−/−^ femur versus the *P4ha2* ^+/−^ control (not statistically significant, *p* = 0.109, Supplemental Fig. S2*A*), whereas BV/TV is significantly reduced in the *P4ha1* ^+/−^; *P4ha2* ^−/−^ proximal tibia versus the *P4ha2* ^+/−^ control (Supplemental Fig. S2*B*) due to a lower trabecular thickness (Tb.Th) (Supplemental Fig. S2*B*). However, at 3 months of age, BV/TV is reduced on average by 65% and 57% in the distal *P4ha1* ^+/−^; *P4ha2* ^−/−^ femur (Fig. 2*B*) and proximal *P4ha1* ^+/−^; *P4ha2* ^−/−^ tibia (Fig. 2*C*), respectively, due to a reduced number of trabeculae (Tb.N) and increased trabecular spacing (Tb.Sp) with representative 3D reconstructions shown of the trabeculae in the proximal tibia (Fig. 2*D*). Of note, inactivation of the C‐P4H‐II isoenzyme alone (ie, *P4ha2* ^−/−^ mice) is sufficient to reduce trabecular BV/TV and Tb.N in the proximal tibia in a statistically significant manner at 3 months (Fig. 2*C*) but not at 6 weeks of age (Supplemental Fig. S2*B*).

**Fig. 3:**
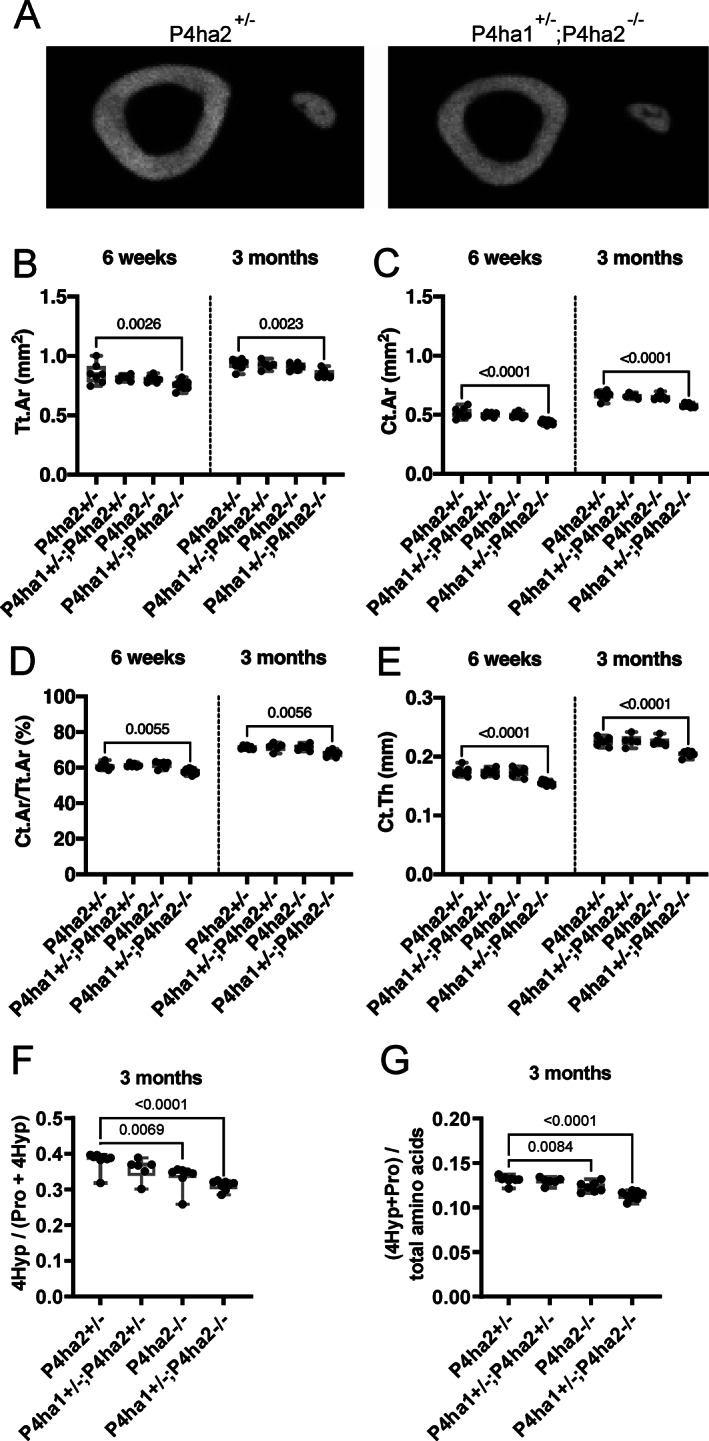
Reduced cortical thickness and collagen hydroxylation degree and amount in the tibias of *P4ha1* ^+/−^; *P4ha2* ^−/−^ mice. (*A*) Representative μCT‐generated images of control *P4ha2* ^+/−^ (left) and *P4ha1* ^+/−^; *P4ha2* ^−/−^ (right) tibial midshaft at 3 months of age. (*B–E*) Quantification of total cross‐sectional area (Tt.Ar) (*B*), cortical bone area (Ct.Ar) (*C*), cortical area fraction (Ct.Ar/Tt.Ar) (*D*), and average cortical thickness (Ct.Th) (*E*) at 6 weeks and 3 months of age. (*F*) Amino acid analysis of the hydroxylation degree of prolines and (*G*) the amount of 4Hyp and Pro per total amino acid count as indicative of collagen content. The data in *B–G* are shown as box and whisker plots, including all individual data points, median, and interquartile range (25th to 75th percentile). Statistical analysis was done with one‐way ANOVA followed by post hoc Dunnett's multiple comparisons test against the control *P4ha2* ^+/−^ mice, *n* = 5–9 mice/genotype. Statistically significant *p* values are shown in the graphs.

Next, we quantified cortical parameters at midshaft of the tibias by μCT (Figs. 2*A* and 3*A*). At both time points, the total cross‐sectional area (Tt.Ar) of the tibias was significantly smaller in the *P4ha1* ^+/−^; *P4ha2* ^−/−^ mice versus the *P4h2* ^+/−^ control mice (Fig. 3*B*). Cortical bone area (Ct.Ar) demonstrated a decrease in the *P4ha1* ^+/−^; *P4ha2* ^−/−^ mice versus the *P4ha2* ^+/−^ control mice at 6 weeks of age, and the difference persisted at 3 months (Fig. 3*C*). Finally, the cortical area fraction (Ct.Ar/Tt.Ar) and cortical thickness (Ct.Th) were significantly decreased in the *P4ha1* ^+/−^; *P4ha2* ^−/−^ mice at both time points versus the *P4ha2* ^+/−^ control mice (Fig. 3*D*, *E*).

### 3.3 Reduced collagen amount but no overt qualitative changes in collagen fibril composition or alignment in the bone ECM

To analyze the collagen hydroxylation degree and collagen amount in tibia, amino acid composition of crude tissue was determined. Analysis of the proline hydroxylation ratio 4Hyp/(4Hyp + Pro) showed, as expected, an allele dose‐dependent reduction in the degree of hydroxylation in *P4ha2* ^−/−^ and *P4ha1* ^+/−^; *P4ha2* ^−/−^ mice (Fig. 3*F*). In addition, as collagen is the major structural protein of connective tissues and is rich in Pro and 4Hyp (the latter being almost exclusively present in collagen), the 4Hyp + Pro/total amino acid ratio reflects the amount of collagen. The results showed that the collagen amount was significantly reduced in both *P4ha2* ^−/−^ and *P4ha1* ^+/−^; *P4ha2* ^−/−^ mice (Fig. 3*G*).

In addition to the observed quantitative changes in collagen, we explored possible qualitative changes in the collagen fibril composition and alignment in the *P4ha1* ^+/−^; *P4ha2* ^−/−^ tibia ECM by SHG microscopy. We first determined the SHG directional forward SHG/backward SHG emission ratios across all genotypes (Supplemental Materials and Methods). This SHG emission ratio is reflective of the fibril diameter, the packing density, and regularity.^(^ ^36^, ^37^ ^)^ Previously, it has been shown that an increase in the type I/type III collagen ratio results in a decreased SHG intensity.^(^ ^36^, ^37^ ^)^ Collagen volume fraction was determined by measuring the mean signal per ROI from both channels. No significant differences were observed between the genotypes, suggesting that C‐P4H deficiency does not affect the type I/type III collagen molecule ratio (Supplemental Fig. S3*A*).

Next, we conducted a directionality analysis on the forward SHG images to analyze the orientation of the collagen fibrils in the *P4ha1* ^+/−^; *P4ha2* ^−/−^ mouse tibias. The type I collagen fibrils are aligned in the plane of the image and have a well‐defined preferred orientation, although some individual fibers are oriented randomly (Supplemental Fig. S3*B*). This localized disarrangement of collagen fibrils seems to be an integral part of the tibia structure. Angular dispersion, which represents the standard deviation of the gaussian curve, shows that no phenotypical differences in the direction of the collagen fibrils between the genotypes are observed (Supplemental Fig. S3*C*). This implies that C‐P4H deficiency does not alter the direction of the fibrils in the tibias. There is some variation in the direction of the fibrils and additional texture parameters within each genotype (Supplemental Figs. S3*C* and S4*A–E*), which may affect the statistical analyses. Altogether, these data indicate that the collagen quantity, but not the collagen fibril quality, is affected in an allele dose‐dependent manner in the mutant mice. These findings are in line with the tight collagen quality control prohibiting secretion of severely underhydroxylated fibril‐forming collagen molecules.^(^ ^28^ ^)^

### 3.4 The *P4ha1* ^+/−^; *P4ha2* ^−/−^ femoral neck is significantly weaker

**Fig. 4:**
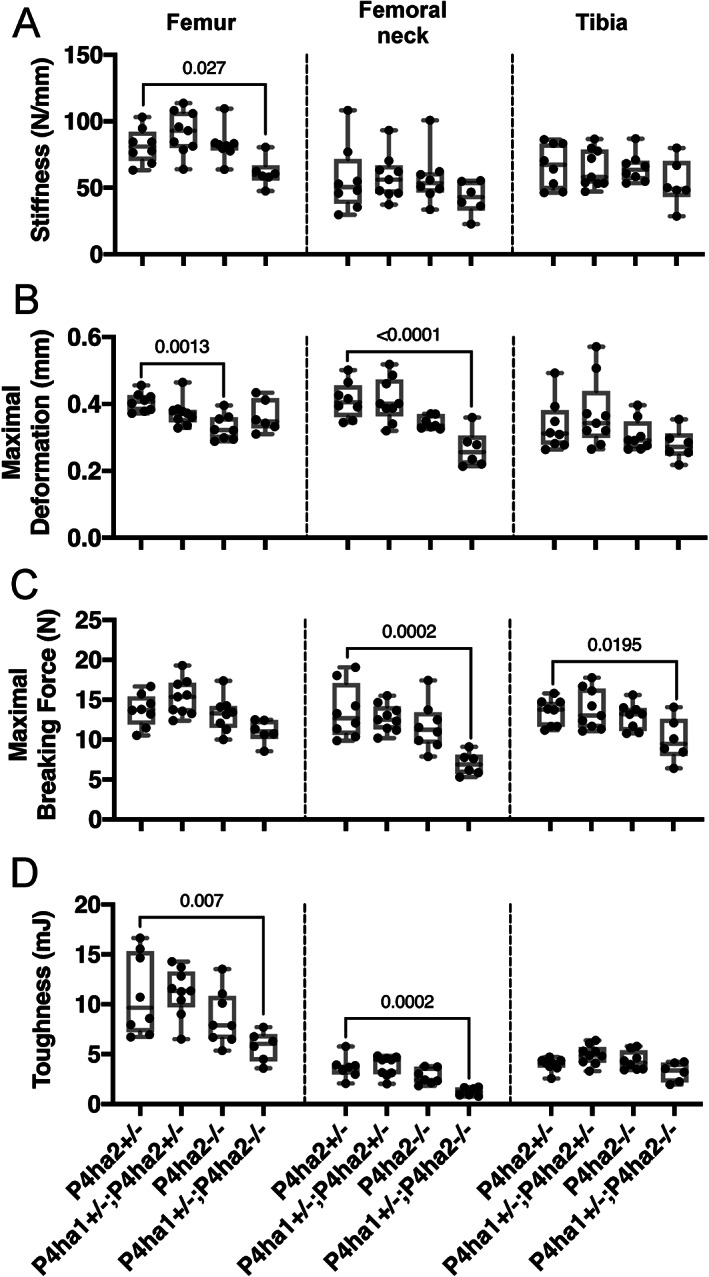
The *P4ha1* ^+/−^; *P4ha2* ^−/−^ femoral neck is significantly weaker on three‐point bending. Three‐point bending of the femur at two locations (midshaft and femoral neck) and the tibia at midshaft. (*A–D*) Quantification of stiffness (N/mm) (*A*), maximal deformation (mm) (*B*), maximal breaking force (N) (*C*), and toughness (mJ) (*D*) at 6 weeks of age. The data are shown as box and whisker plots including all individual data points, median, and interquartile range (25th to 75th percentile). Statistical analysis was done with one‐way ANOVA followed by post hoc Dunnett's multiple comparisons test against the control *P4ha2* ^+/−^ mice, *n* = 6–9 mice/genotype. Statistically significant *p* values are shown in the graphs. Maximal breaking force (*C*) follows a decreasing trend at the *P4ha1* ^+/−^; *P4ha2* ^−/−^ femoral diaphysis but does not reach statistical significance (*p* = 0.0634 for the *P4ha1* ^+/−^; *P4ha2* ^−/−^ mice versus control).

To measure the strength of the bone matrix at 6 weeks of age, the long bones were subjected to mechanical loading at three different sites by three‐point bending. The stiffness (N/mm) was significantly reduced at the *P4ha1* ^+/−^; *P4ha2* ^−/−^ femoral diaphysis versus the *P4ha2* ^+/−^ control mice but not at the femoral neck or tibia (Fig. 4*A*). A lower maximal deformation was observed at the femoral diaphysis of the *P4ha2* ^−/−^ mice and at the femoral neck of the *P4ha1* ^+/−^; *P4ha2* ^−/−^ versus the *P4ha2* ^+/−^ control mice (Fig. 4*B*). The maximal breaking force (N) was significantly lower in the *P4ha1* ^+/−^; *P4ha2* ^−/−^ femoral neck and, to a lesser extent, in the *P4ha1* ^+/−^; *P4ha2* ^−/−^ tibia versus the *P4ha2* ^+/−^ control mice (Fig. 4*C*). Toughness, or the work required for fracture, was significantly lower in the *P4ha1* ^+/−^; *P4ha2* ^−/−^ femoral diaphysis and femoral neck versus the *P4ha2* ^+/−^ control mice (Fig. 4*D*). These properties were also noted subjectively during the preparation of the bone samples as the *P4ha1* ^+/−^; *P4ha2* ^−/−^ femoral neck was the most fragile site. The *P4ha2* ^−/−^ bones were unremarkable during preparation. Of note, due to the rapidly changing geometry along the diaphysis of the tibia,^(^ ^50^ ^)^ three‐point bending may not be sufficient to delineate differences in bone strength of the tibias between the genotypes used in this study.

### 3.5 Lower osteoid fraction in the proximal *P4ha1* ^+/−^; *P4ha2* ^−/−^ tibia

Because the μCT analysis showed a pronounced osteopenic phenotype in the *P4ha1* ^+/−^; *P4ha2* ^−/−^ mice (Supplemental Fig. S2*B* and Fig. 2*C*), while no overt changes in the composition of the bone ECM were found on SHG microscopy (Supplemental Fig. S3), we performed static histomorphometric analyses of Masson‐Goldner's trichrome and TRAP‐stained samples of the proximal tibia at 6 weeks and 3 months of age (Supplemental Fig. S5). BV/TV in the static histomorphometric analyses (Supplemental Fig. S5*C, D*) correlated with the BV/TV observed by μCT (Fig. 2*C*; Supplemental Fig. S2*B*), especially at 6 weeks of age with representative images shown in Supplemental Fig. S5*E*, *F*.

**Fig. 5:**
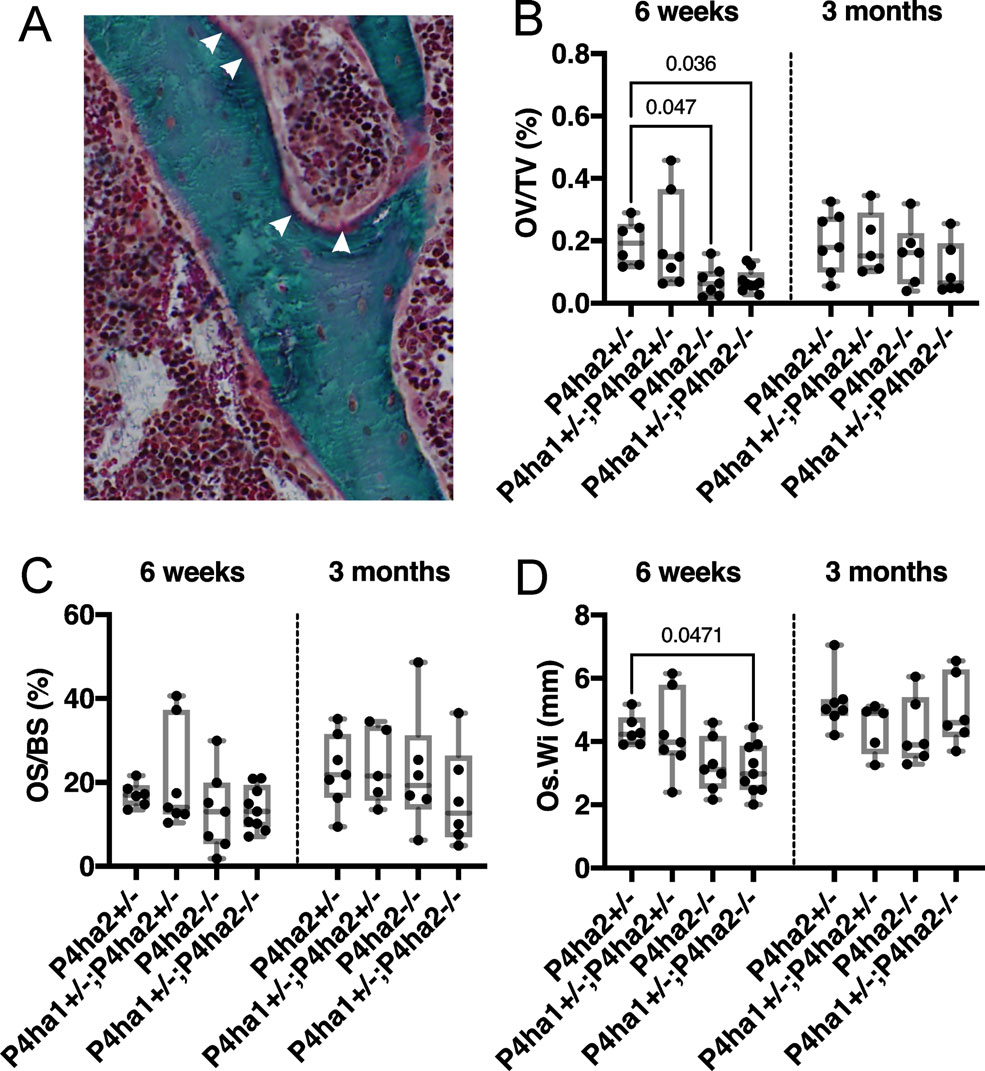
Reduced osteoid fraction in the proximal *P4ha1* ^+/−^; *P4ha2* ^−/−^ tibias at 6 weeks of age. (*A*) A Masson‐Goldner's trichrome‐stained trabecula in the proximal tibia showing mineralized ECM in green and unmineralized ECM in red (white arrowheads) at 6 weeks of age. (*B–D*) Quantification of osteoid volume/tissue volume (OV/TV) (*B*), osteoid surface (OS/BS) (*C*), and osteoid width (Os.Wi) (*D*) in the proximal tibia at 6 weeks and 6 months of age. The data in *B–D* are shown as box and whisker plots including all individual data points, median, and interquartile range (25th to 75th percentile). Statistical analysis was done with one‐way ANOVA followed by post hoc Dunnett's multiple comparisons test against the control *P4ha2* ^+/−^ mice, *n* = 5–9 mice/genotype. Statistically significant *p* values are shown in the graphs.

The Masson‐Goldner's trichrome staining shows the mineralized ECM in green and unmineralized ECM (ie, osteoid) in red (Fig. 5*A*, white arrowheads). The osteoid fraction (OV/TV) was significantly reduced in the *P4ha2* ^−/−^ and *P4ha1* ^+/−^; *P4ha2* ^−/−^ mice at 6 weeks but not at 3 months of age versus the *P4ha2* ^+/−^ control mice (Fig. 5*B*). Furthermore, when the osteoid surface was normalized over existing bone surface (OS/BS), no differences were observed in any genotype at either time point (Fig. 5*C*). A reduced osteoid width (Os.Wi) was observed in the *P4ha1* ^+/−^; *P4ha2* ^−/−^ mice versus the *P4ha2* ^+/−^ control mice at 6 weeks of age (Fig. 5*D*).

### 3.6 Slower trabecular bone formation in the proximal *P4ha1* ^+/−^; *P4ha2* ^−/−^ tibia

**Fig. 6:**
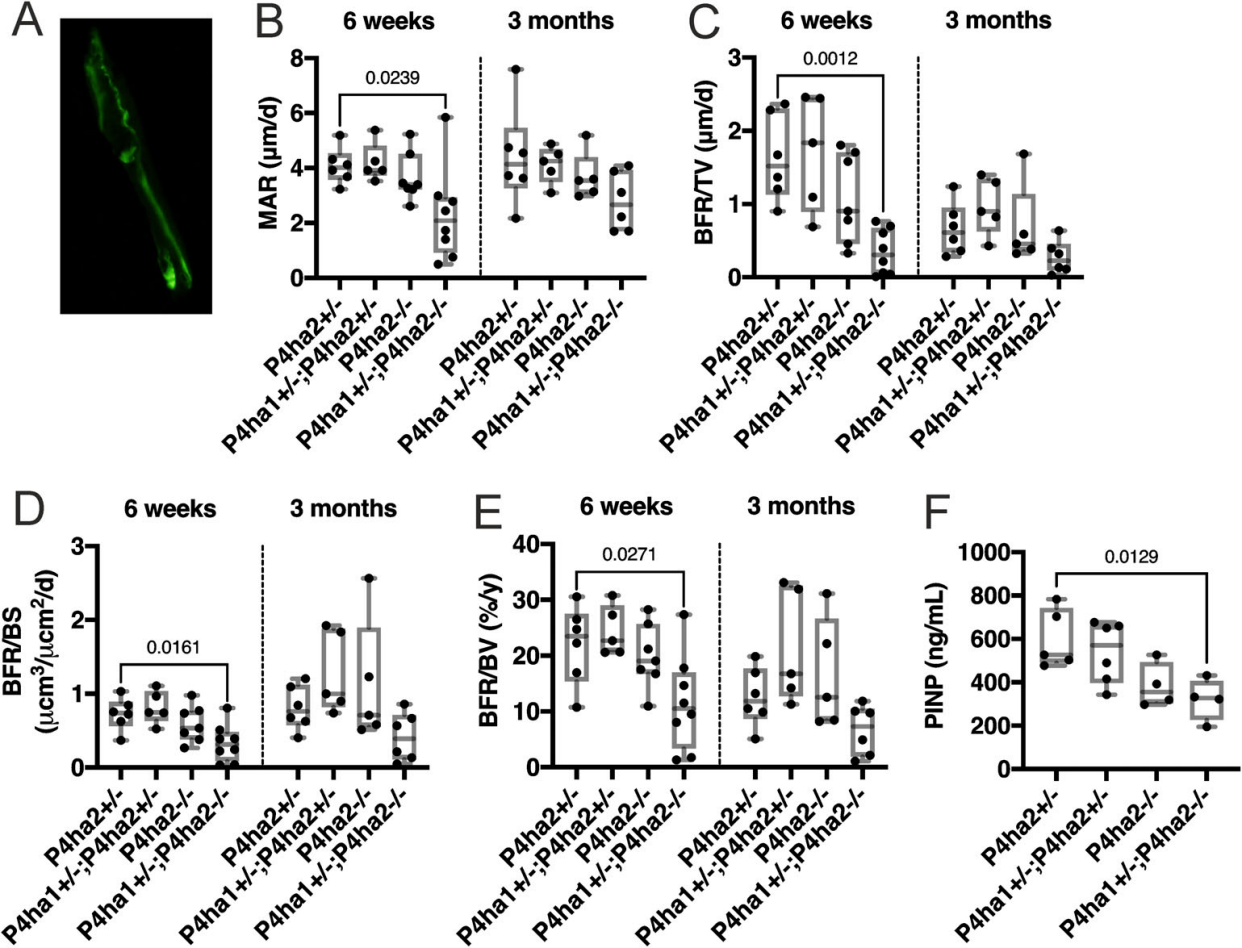
The trabecular bone formation rate is reduced in the *P4ha1* ^+/−^; *P4ha2* ^−/−^ tibias at 6 weeks but not at 3 months of age. (*A*) Calcein fluorescence signal in the trabecular matrix. (*B–E*) Quantification of mineral apposition rate (MAR) (*B*), bone formation rate/tissue volume (BFR/TV) (*C*), bone formation rate/bone surface (BFR/BS) (*D*), and bone formation rate/bone volume (BFR/BV) (*E*) in the proximal tibia at 6 weeks and 3 months of age. (*F*) Serum procollagen type I N propeptide (PINP) at 5 weeks of age. The data in *B–F* are shown as box and whisker plots including all individual data points, median, and interquartile range (25th to 75th percentile). Statistical analysis was done with one‐way ANOVA followed by post hoc Dunnett's multiple comparisons test against the control *P4ha2* ^+/−^ mice, *n* = 4–8 mice/genotype. Statistically significant *p* values are shown in the graphs.

Next, to perform dynamic histomorphometric analyses of the tibias, the mice were injected intraperitoneally with fluorescent calcein at 6 and 2 days before euthanization and visualized by fluorescence microscopy (Fig. 6*A*). There was a significant decrease in the mineralizing surface (MS) and the single‐labeled surface (sLS) in the *P4ha1* ^+/−^; *P4ha2* ^−/−^ tibia at 6 weeks but not at 3 months of age versus the *P4ha2* ^+/−^ control mice (Supplemental Fig. S6*A*, *B*). Double‐labeled surface (dLS) followed a decreasing trend whereby dLS in the *P4ha1* ^+/−^; *P4ha2* ^−/−^ samples was half of that in the *P4ha2* ^+/−^ control mice, but the difference did not reach statistical significance (Supplemental Fig. S6*C*). Finally, when the MS was normalized over existing bone surface (MS/BS), there was a significant decrease in the *P4ha1* ^+/−^; *P4ha2* ^−/−^ tibia at 6 weeks but not at 3 months of age versus the *P4ha2* ^+/−^ control mice (Supplemental Fig. S6*D*).

The mineral apposition rate (MAR) was reduced in the *P4ha1* ^+/−^; *P4ha2* ^−/−^ tibia versus the *P4ha2* ^+/−^ control mice at 6 weeks but not at 3 months of age (Fig. 6*B*). Subsequently, the bone formation rate (BFR) normalized over three different referents (ie, BFR/TV, BFR/BS, and BFR/BV) was significantly reduced in the *P4ha1* ^+/−^; *P4ha2* ^−/−^ tibia versus the *P4ha2* ^+/−^ control mice at 6 weeks of age (Fig. 6*C*–*E*). A decreasing trend in the BFR was observed in the *P4ha1* ^+/−^; *P4ha2* ^−/−^ tibia at 3 months of age, but it did not reach statistical significance (Fig. 6*C*–*E*). MAR at the periosteum and endosteum (Supplemental Fig. S7*A*) was not changed at either time point (Supplemental Fig. S7*B*), despite the differences in the cortical bone parameters reported above (Fig. 3).

To approximate the rate of bone matrix production before the 6‐week time point, we collected serum samples at 5 weeks of age and quantified the concentration of the procollagen type I N‐terminal propeptide (PINP). PINP is considered a reliable biomarker for bone formation and mineral apposition rates.^(^ ^51^ ^)^ The serum concentration of PINP was significantly lower in the *P4ha1* ^+/−^; *P4ha2* ^−/−^ mice versus the *P4ha2* ^+/−^ control mice, and an apparent allele‐dose‐dependent manner was observed (Fig. 6*F*).

Finally, the fraction of osteoid undergoing mineralization (MS/OS) followed a decreasing trend in the *P4ha1* ^+/−^; *P4ha2* ^−/−^ mice versus the *P4ha2* ^+/−^ control mice at 6 weeks of age (Supplemental Fig. S8), but the difference did not reach statistical significance on one‐way ANOVA (*p* = 0.164 on one‐way ANOVA followed by post hoc Dunnett's multiple comparisons test).

### 3.7 A smaller population of and lower C‐P4H activity in the *P4ha1* ^+/−^; *P4ha2* ^−/−^ osteoblasts

**Fig. 7:**
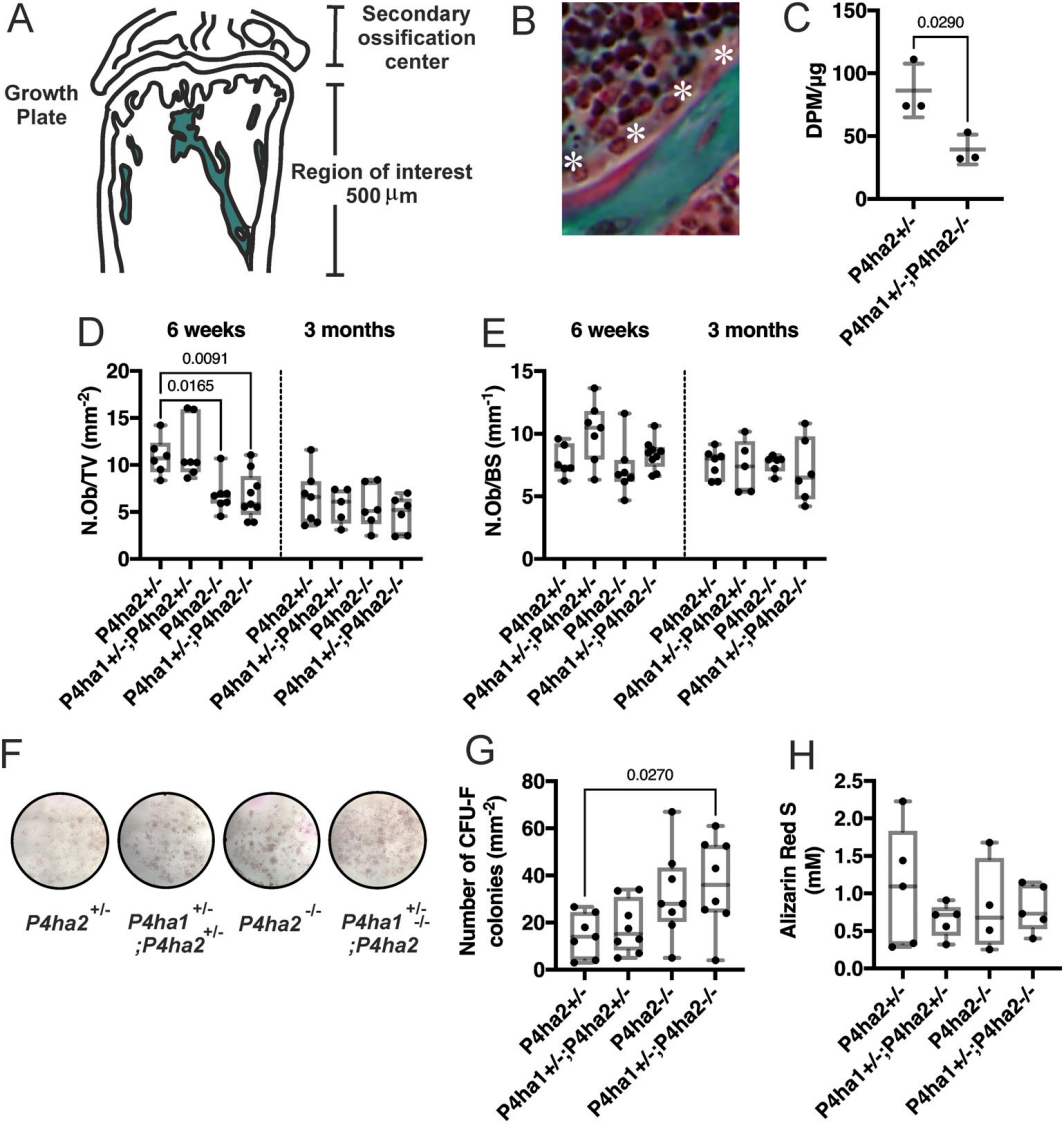
The number of osteoblasts and their C‐P4H activity are reduced in the *P4ha1* ^+/−^; *P4ha2* ^−/−^ tibias. (*A*) A schematic drawing defining the region of interest for the static histomorphometric analyses. (*B*) A Masson‐Goldner's trichrome‐stained trabecula in the proximal tibia showing osteoblasts (white asterisks) as cuboidal mononuclear cells adjacent to the bone ECM at 6 weeks of age. (*C*) C‐P4H activity in osteoblasts isolated from tibias and femurs at 5 weeks of age. (*D*, *E*) Quantification of osteoblast number/tissue volume (N.Ob/TV) and osteoblast number/bone surface (N.Ob/BS) in the proximal tibia at 6 weeks and 3 months of age. (*F–H*) Bone marrow cells flushed from both tibias and femurs at 5 weeks of age were seeded on 6‐well plates at 16 × 10^6^ cells per well to isolate the colony‐forming unit‐fibroblasts (CFU‐F). To promote ECM production, the CFU‐F colonies were incubated in osteogenic media, including ascorbic acid and glycerol 2‐phosphate. (*F*) Representative images of CFU‐F colonies stained for alkaline phosphatase (ALP). (*G*) Number of ALP‐positive CFU‐F colonies/mm^−2^. (*G*) Quantification of the Alizarin Red S‐stained mineralized matrix produced by the CFU‐F after stimulation in vitro. The data in *C–H* are shown as box and whisker plots including all individual data points, median, and interquartile range (25th to 75th percentile), *n* = 3–9 mice/genotype. Statistical analysis was done with one‐way ANOVA followed by post hoc Dunnett's multiple comparisons test against the control *P4ha2* ^+/−^ mice, where the number of groups was three or more; two‐tailed Student's *t* test was used to compare two groups (*C*). Statistically significant *p* values are shown in the graphs.

The trabecular region of interest was defined as the area 500 μm in height below the growth plate, excluding the growth plate, the growth plate osteoblasts, and the cortical bone and cortical osteoblasts (Fig. 7*A*). The osteoblasts were defined as cuboidal mononuclear cells that lie adjacent to the bone ECM (Fig. 7*B*, white asterisks).

Despite the ubiquitous expression of *P4ha1* and *P4ha2* in the adult murine bone marrow (Supplemental Fig. S1), previous lineage‐tracing studies^(^ ^52^ ^)^ and the expression analysis carried out here (Supplemental Fig. S1) show that osteoblasts, and not endothelial cells or mural cells, are responsible for the production of the type I collagen present in the bone ECM.^(^ ^52^ ^)^ To measure the total C‐P4H activity in the osteoblasts, we isolated mature osteoblasts from the long bones of control and *P4ha1* ^+/−^; *P4ha2* ^−/−^ mice as described. Nonidet P‐40‐soluble lysates were used as source for the enzyme, and [^14^C]proline‐labeled chick type I procollagen α chains as substrate. The total C‐P4H activity in *P4ha1* ^+/−^; *P4ha2* ^−/−^ osteoblasts was approximately 46% of that of the control *P4ha2* ^+/−^ osteoblasts (Fig. 7*C*), which is in line with similar measurements performed on growth plate chondrocytes.^(^ ^29^ ^)^ We also measured the C‐P4H activity in the double heterozygous and *P4ha2* ^−/−^ osteoblasts, which showed 92% and 94% activity relative to the control, respectively (Supplemental Fig. S9), in line with previous findings in chondrocytes^(^ ^29^ ^)^ and the current data that C‐P4H‐I can to a large extent compensate for the lack of C‐P4H‐II and a marked effect on the C‐P4H activity and phenotype is observed only when the lack of C‐P4H‐II is combined with haploinsufficiency of C‐P4H‐I. These results are also in agreement with the reduced proline hydroxylation ratio and reduced collagen amount (Fig. 3*F*, *G*).

To compensate for the reduced C‐P4H activity, we expected to observe a larger pool of bone‐forming osteoblasts (N.Ob/TV) in the trabecular region. However, the *P4ha1* ^+/−^; *P4ha2* ^−/−^ mice, as well as *P4ha2* ^−/−^ mice, presented with a smaller N.Ob/TV at 6 weeks of age versus the *P4ha2* ^+/−^ control mice (Fig. 7*D*). The size of the osteoblast population in the *P4ha2* ^−/−^ and *P4ha1* ^+/−^; *P4ha2* ^−/−^ mice had reached a similar level at 6 weeks of age that was found in the 3‐month‐old mice across all four genotypes (Fig. 7*D*). When the N.Ob was normalized over existing bone surface (N.Ob/BS), no differences were observed at either time point (Fig. 7*E*).

Next, to assess the production of ECM by the *P4ha1* ^+/−^; *P4ha2* ^−/−^ osteoblasts, we isolated the colony‐forming unit‐fibroblasts (Fig. 7*F*). The CFU‐F represent a heterogenous cell population that includes skeletal stem cells and osteoprogenitors that are able to differentiate into osteoblast progenitors and mature osteoblasts under proper in vitro culture conditions.^(^ ^53^, ^54^, ^55^ ^)^ In contrast to the smaller population of the bone‐forming osteoblasts in the *P4ha1* ^+/−^; *P4ha2* ^−/−^ mice versus the *P4ha2* ^+/−^ control mice, the number of positive CFU‐F colonies showed an allele‐dose‐dependent increase and was doubled in the *P4ha1* ^+/−^; *P4ha2* ^−/−^ mice (Fig. 7*G*). However, despite the larger CFU‐F population, the amount of mineralized ECM produced by the *P4ha1* ^+/−^; *P4ha2* ^−/−^ cells after stimulation was equal to the amount of ECM produced by the *P4h2* ^+/−^ control cells (Fig. 7*H*).

### 3.8 Osteoclastogenesis is coupled to the osteoblast pool

**Fig. 8:**
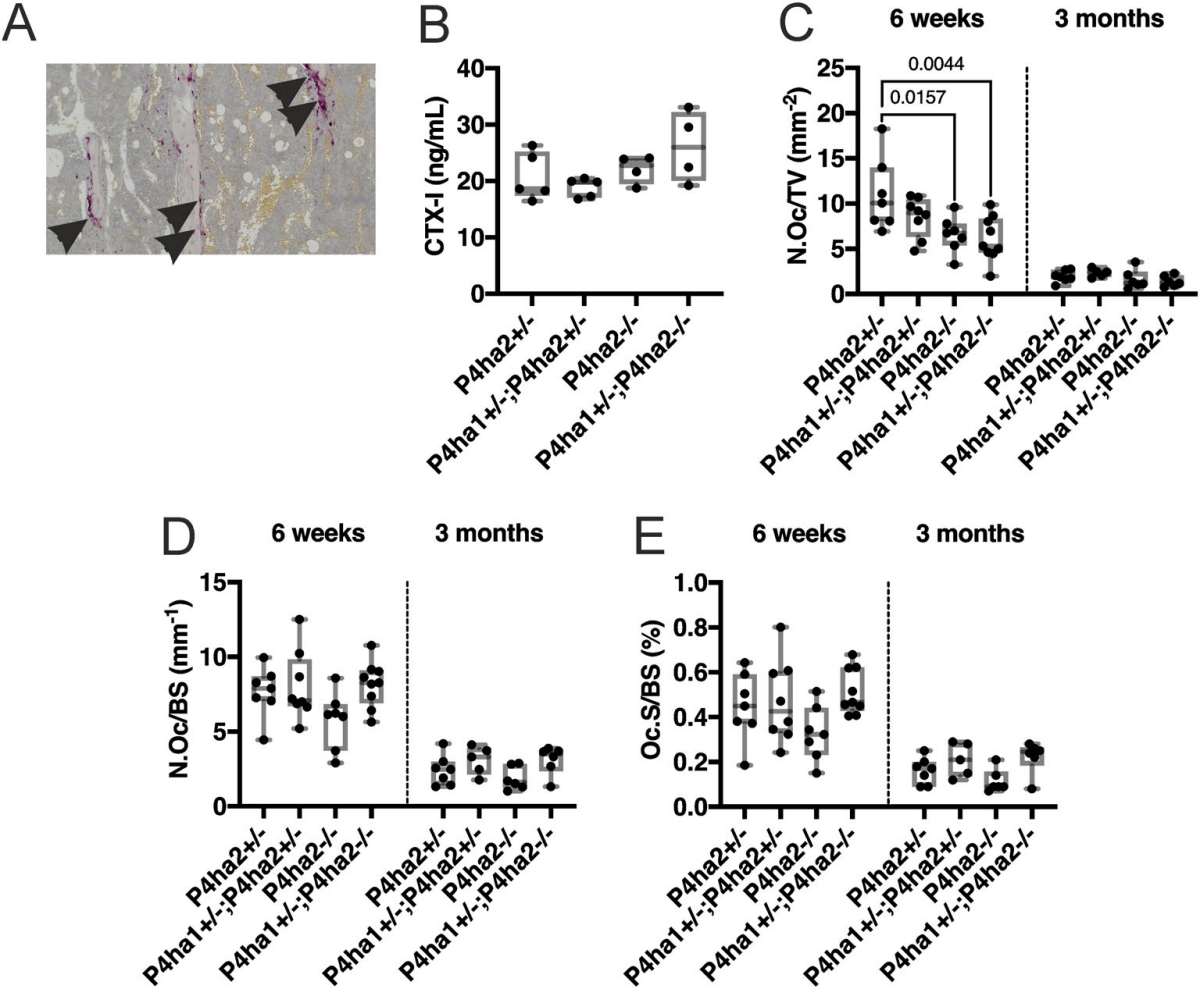
Reduced number of osteoclasts (N.Oc/TV) in the *P4ha1* ^+/−^; *P4ha2* ^−/−^ mice at 6 weeks of age. (*A*) TRAP‐stained osteoclasts (black arrowheads) adjacent to trabeculae. (*B*) Serum cross‐linked C‐telopeptide of type I collagen (CTX‐I) at 5 weeks of age. (*C–E*) Quantification of osteoclast number/tissue volume (N.Oc/TV) (*C*), osteoblast number/bone surface (N.Oc/BS) (*D*), and osteoclast surface/bone surface (Oc.S/BS) (*E*) at 6 weeks and 3 months of age. The data in *B–E* are shown as box and whisker plots including all individual data points, median, and interquartile range (25th to 75th percentile). Statistical analysis was done with one‐way ANOVA followed by post hoc Dunnett's multiple comparisons test against the control *P4ha2* ^+/−^ mice, *n* = 5–9 mice/genotype. Statistically significant *p* values are shown in the graphs.

Finally, we investigated the role of the matrix‐resorbing osteoclasts (Fig. 8 *A*, black arrowheads) in producing the *P4ha1* ^+/−^; *P4ha2* ^−/−^ bone phenotype. To quantify the rate of bone ECM degradation, we collected serum samples at 5 weeks of age and measured the serum concentration of CTX‐I, a biomarker for ECM resorption by the osteoclasts.^(^ ^51^ ^)^ The serum CTX‐I concentrations were unchanged across all genotypes (Fig. 8*B*).

As the coordination of osteoclast differentiation and bone turnover is coupled to the osteoblast population via the receptor activator of NF‐κB ligand (RANKL)‐osteoprotegerin (OPG) signaling pathway,^(^ ^56^ ^)^ we hypothesized that the osteoclast number would correlate with the osteoblast number. In line with our hypothesis, the total osteoclast number (N.Oc/TV) was reduced significantly in the *P4ha2* ^−/−^ and *P4ha1* ^+/−^; *P4ha2* ^−/−^ tibia at 6 weeks but not at 3 months of age versus the *P4ha2* ^+/−^ control mice (Fig. 8*C*). Furthermore, N.Oc/TV decreased with age (Fig. 8*C*). When the N.Oc was normalized over existing bone surface (N.Oc/BS), no differences were observed in any of the genotypes at either time point (Fig. 8*D*). Again, the N.Oc/BS was reduced with age in the 3‐month‐old mice (Fig. 8*D*). The results remained the same when measured as osteoclast surface over existing bone surface (Oc.S/BS) (Fig. 8*E*).

## 4 Discussion

We have generated the transgenic *P4ha1* ^+/−^; *P4ha2* ^−/−^ mouse line to study the role of C‐P4H activity in mouse development because a homozygous deletion of *P4ha1* is embryonic lethal at E10.5 and the *P4ha2* ^−/−^ mice present with very minor phenotypic changes, suggesting an allele‐dose‐dependent importance for the developing organism.^(^ ^28^, ^29^ ^)^ By knocking out one allele of *P4ha1* and both alleles of *P4ha2*, we achieved about 50% decrease in the total C‐P4H activity in osteoblasts, which is close to the about 65% decrease in chondrocytes.^(^ ^29^ ^)^ A markedly larger decrease in the C‐P4H activity, about 80%, is observed in *P4ha1* ^−/−^ fibroblasts and embryos, resulting in lethality.^(^ ^28^, ^29^ ^)^ We have previously shown that the *P4ha1* ^+/−^; *P4ha2* ^−/−^ mice are smaller than their littermates and develop shorter long bones and chondrodysplasia due to a transient inner cell death phenotype of the developing growth plate. Here, we show a lower number of trabeculae (Fig. 2), a reduced amount of osteoid (Fig. 5), and a reduced amount of collagen (Fig. 3G) but no overt changes in the composition (Supplemental Fig. S3) or the mineralization of the bone ECM (Supplemental Figs. S6 and S8), suggesting that bone matrix production peaks earlier in the *P4ha1* ^+/−^; *P4ha2* ^−/−^ mice. This earlier peak in bone mass accrual, and thus an earlier attainment of homeostasis between bone ECM production and resorption, is further denoted by the number of osteoblasts (N.Ob/TV and N.Ob/BS, Fig. 7), which in the *P4ha1* ^+/−^; *P4ha2* ^−/−^ mice at 6 weeks of age is similar to all genotypes at 3 months of age. Our present data on C‐P4H α subunit expression, which appears to be highest before the 2‐week time point (Fig. 1) further implies a crucial role very early on. However, despite the smaller pool of osteoblasts, there appears to be a larger population of CFU‐F cells in the *P4ha1* ^+/−^; *P4ha2* ^−/−^ bone marrow. The precise identity of these cells and detailed implications of this finding warrant further investigations, including quantification of mesenchymal/osteoprogenitor cell and osteoblast markers (eg, osterix) by immunohistochemistry. Similarly, it would be interesting to study the phenotype in aged mice to determine whether the osteopenia is resolved as the osteoblast numbers normalize.

The C‐P4Hs catalyze the formation of 4Hyp in the ‐X‐Pro‐Gly‐ repeats of all known collagen types and more than 20 proteins with collagen‐like domains, where the 4Hyp is necessary for the folding and thermal stability of the collagen triple helix.^(^ ^1^ ^)^ Although we have previously shown that inappropriate hydroxylation results in an abnormal deposition and structure of the ECM and impairs integrin signaling,^(^ ^29^, ^57^ ^)^ this process is far from being a passive structural modification but regulates the interactive alignment between cells and the ECM. For example, osteoblasts orientate themselves according to the collagen fibrils that they secrete, a process that enhances the ability of the ensuing osteocyte to respond to the mechanical loading that runs through the ECM.^(^ ^58^, ^59^ ^)^ In addition, patient data and in vivo cancer models suggest that C‐P4Hs regulate collagen alignment, which contributes to the metastatic process in breast cancer and melanoma, and that the inhibition of C‐P4H activity suppresses metastasis.^(^ ^14^, ^15^, ^16^, ^60^ ^)^ To add another layer of complexity, the C‐P4Hs have been shown to regulate and respond to changes in cell metabolism via epigenetic changes and the stabilization of the hypoxia‐inducible factors by consuming 2‐oxoglutarate, molecular oxygen, and vitamin C.^(^ ^11^, ^12^, ^13^ ^)^ Our present study shows that C‐P4H activity is a fundamental feature of the osteoblasts and that these cells provide an accessible platform to elucidate the complex role of C‐P4Hs in cellular physiology in the future.

To better define the mechanisms involved in our osteopenic phenotype, future studies of the *P4ha1* ^+/−^; *P4ha2* ^−/−^ mouse model should include a comparison of bones formed by endochondral ossification and the intramembranous bones,^(^ ^61^ ^)^ which are not affected by the chondrodysplasia observed in these mice. An osteopenic phenotype in the intramembranous bones would support an osteoblast cell‐autonomous mechanism and downplay the role of chondrocytes. Similarly, further mouse models with conditional inactivation of the C‐P4H α subunit genes, *P4ha1*, *P4ha2*, or *P4ha3*, in a specific cell type (eg, osteoblasts or chondrocytes) would be valuable to minimize the impact of neighboring cell types and tissues and facilitate a better understanding of the need and individual functions of the three C‐P4H isoenzymes. For example, C‐P4H‐I and C‐P4H‐II show distinct differences in their peptide substrate *K*_*m*_ values and inhibition of substrate binding, and the biological meaning of these differences remains unknown.^(^ ^2^ ^)^ Here, we show that inactivation of the *P4ha2* gene and, thus, functional C‐P4H‐II, is sufficient to produce small, but significant, differences (Figs. 2*C*, 4*B*, 5*B*, 7*D*, and 8*C*) without affecting the total C‐P4H activity as measured using type I procollagen α chains as substrate (Supplemental Fig. S9). This could imply that C‐P4H‐II targets different collagen substrates present in the bone ECM, and such differences in substrate specificities should be explored in the future. With regard to C‐P4H‐III, our data show a high expression of the *P4ha3* gene in the early growth plate at P0 and the myeloid‐supportive O1 cluster of osteoblasts in the adult mice (Fig. 1 and Supplemental Fig. S1), possibly implying important roles in the embryo and a myeloid‐supportive role in the adult mouse.

The value of our current *P4ha1* ^+/−^; *P4ha2* ^−/−^ mouse model is that it replicates key aspects of the recently recognized congenital connective tissue disorder caused by elaborate biallelic pathogenic variants of the human *P4HA1* gene, namely mild growth restriction and bone dysplasia.^(^ ^31^ ^)^ Interestingly, the two proband *P4HA1* alleles carried distinct pathogenic variants (ie, one frameshift and one splice site), whereby wild‐type mRNA was produced only from the intact transcript that resulted from mutually exclusive alternative splicing. The total C‐P4H activity in the proband skin fibroblasts was approximately 50% of the age‐matched control fibroblasts and the amount of 4Hyp and thermal stability of secreted collagen was slightly reduced. Because the *P4ha1* ^−/−^ mouse is embryonic lethal, it is straightforward to predict that a homozygous loss‐of‐function of human *P4HA1* would be too. These findings could explain why *P4HA1*‐associated disorders are so rare, whereas *P4HA2*‐associated disorders present with a mild phenotype, mainly high myopia.^(^ ^32^ ^)^ Nevertheless, the *P4ha1* ^+/−^; *P4ha2* ^−/−^ mouse model may be useful in screening for potential treatment options, such as bisphosphonates and newer anabolic drugs,^(^ ^62^ ^)^ in disease caused by pathogenic variants of the *P4HA1* gene.

Finally, as described above, the C‐P4Hs are of current medical interest because they have been shown to be involved in the metastatic process and regulate ECM formation in not only healthy tissues but also in diseases such as pulmonary and kidney fibrosis, hepatic cirrhosis, and systemic sclerosis.^(^ ^2^, ^16^, ^63^, ^64^ ^)^ In addition, the C‐P4H reaction mechanism is common to other, closely related 2‐OGDDS, such as the HIF‐P4Hs, and is thus sensitive to off‐target inhibition.^(^ ^10^, ^11^ ^)^ Our data demonstrate that C‐P4H activity is necessary for bone mass accrual during development and that a deficiency of C‐P4H activity is associated with a reduced bone formation rate (Fig. 6*C*–*E*). Although many C‐P4H inhibitors have been identified, none are effective and selective enough to warrant long‐term application in human patients.^(^ ^2^, ^63^ ^)^ Nevertheless, based on the current results, we regard it important that potential C‐P4H inhibitors, or drugs that may result in off‐target inhibition, should be screened for side effects related to the bone ECM, and that these adverse effects should be investigated further using the C‐P4H mouse models.

In conclusion, we demonstrate that the highest mRNA expression of the C‐P4H α subunits is observed before the 2‐week time point in the murine long bones and that *P4ha1* is the predominant C‐P4H α subunit. Furthermore, we show that a 50% relative reduction in total C‐P4H activity and concomitant reduction in 4Hyp coupled with reduced collagen amount in the *P4ha1* ^+/−^; *P4ha2* ^−/−^ mouse model results in a significant loss of bone mass and strength in the long bones and that the osteopenia is more evident in the proximal tibia, whereas the femoral neck is the weakest site on three‐point bending. However, there are no overt qualitative changes in the matrix composition across the four genotypes, implying that the phenotype found in the *P4ha1* ^+/−^; *P4ha2* ^−/−^ mice arises due to slower and quantitatively compromised matrix production. Our previous data suggest that the *P4ha1* ^+/−^; *P4ha2* ^−/−^ growth plates suffer from transient chondrocyte cell death, which may also explain the earlier plateauing in matrix production in the *P4ha1* ^+/−^; *P4ha2* ^−/−^ mice. Furthermore, we observed a smaller osteoblast population but an increased population of mesenchymal progenitors in the *P4ha1* ^+/−^; *P4ha2* ^−/−^ mice. Finally, we provide a prospective direction for future studies of the C‐P4H enzymes, which carry substantial promise in terms of treatment in cancer and fibrotic disease.

## Disclosures

JM owns equity in FibroGen Inc., which develops P4H inhibitors as potential therapeutics. This company supports research in the JM group. All other authors report no conflicts of interest.

## Author Contributions

**Jussi‐Pekka Tolonen:** Data curation; formal analysis; investigation; methodology; validation; visualization; writing – original draft; writing – review and editing. **Antti M Salo:** Data curation; formal analysis; investigation; methodology; validation; visualization; writing – original draft; writing – review and editing. **Mikko Finnilä:** Formal analysis; investigation; methodology. **Ellinoora Aro:** Formal analysis; investigation. **Emma Karjalainen:** Investigation. **Veli‐Pekka Ronkainen:** Investigation; methodology. **Kati Drushinin:** Investigation. **Christophe Merceron:** Investigation; methodology. **Valerio Izzi:** Investigation. **Ernestina Schipani:** Conceptualization; funding acquisition; methodology; resources; supervision; writing – review and editing. **Johanna Myllyharju:** Conceptualization; funding acquisition; methodology; project administration; resources; supervision; writing – review and editing.

### Peer Review

The peer review history for this article is available at https://publons.com/publon/10.1002/jbm4.10630.

## Supporting information


Appendix S1.

## Data Availability

The data that support the findings of this study are available from the corresponding author upon reasonable request.
