# Supplementary material for: Reduced Bone Mass in Collagen Prolyl 4‐Hydroxylase P4ha1 +/−; P4ha2 −/− Compound Mutant Mice
Source: JBMR Plus. 2022 May 9;6(6):e10630. doi: 10.1002/jbm4.10630 (PMC9189910; doi:10.1002/jbm4.10630)
Supplement: Supplementary file 1 — Appendix S1. [file JBM4-6-e10630-s001.docx]

**Supplement**

**Supplementary Methods – Second Harmonic Generation (SHG) microscopy**

**Sample preparation**

3-month-old female mice were used for the SHG microscopy analyses. Preparation of the 5-μm paraffin slides, which were also used for the histomorphometric assays, is described in the main Materials and Methods.

**SHG microscopy**

SHG imaging of fibrillar collagen was performed by a Nikon A1R MP+ upright multiphoton microscope (Nikon, Japan). Briefly, 820 nm laser wavelength was used for SHG generation, and signals were collected in episcopic (backward) direction by using Apochromat 25x/1.1W MP1300 objective, and in transmitted (forward) direction by using A1-NDN (NA 1.2 W) condenser. 458 nm short pass filters and GaAsP PMTs were used for detecting SHG signals in both directions. The size of the field of view (FOV) was 509 µm x 509 µm, and the pixel size was set to 0.12 µm with 3.1 µs pixel dwell time and 4x line average. The imaged regions were selected consistently three fields of view (3FOV) from the proximal epiphysis of the cortical bone towards the shaft of diaphysis.

**Quantification**

Image analyses were performed using ImageJ (v. 1.51i, National Institute of Health, Bethesda, MD, USA). All slices were produced and imaged in the same way and all analysis setups were the same for all images. Prior to image analysis, the collected stacks were split into their respective forward and backward channels. The forward collected images can reveal both smaller features as well as more disjointed fibrils that result from smaller, more random fibril or fiber structures, where the latter appear adjacent in the backward-detected images (LaComb et al. 2008). Thus, for the directionality and Gray level co-occurrence matrix (GLCM) analyses, we used only the forward channel.

**Backward/forward ratio**

Magic wand tool (tolerance 15, 8-connected) was used to select regions inside the cortex and regions of interest (ROIs) were determined to analyze the cortical bone collagen fibers. For forward/backward analysis, the same threshold was applied to both channels before averaging the total SHG intensity in each optical section (Tilbury et al. 2014). Collagen volume fraction was determined by measuring the mean signal per ROI area form both channels. Forward/backward ratio was then determined by dividing forward measurement by backward measurement. Numerical values of the forward/backward ratio were graphically plotted (**Supplementary Figure S3A**).

**Directionality**

First, an image was processed by application of spatial frequency filters (ImageJ→ Process→ Fourier transform (FFT) → Bandpass filter). ImageJ bandpass filters have the capacity to remove both high and low spatial frequencies. The filter sets for large and small structures were set at 40 and 3 pixels, respectively. Suppression stripes were set to ‘none' and tolerance of direction was set at 5%. ‘Autoscale after filtering' and ‘saturate image when autoscaling' were activated during processing. Then, the filtered images were converted to binary by adjusting the threshold (ImageJ→ Adjust→ Threshold→ Apply). To analyze the mean orientation and the angular dispersion of the collagen fibrils, directionality analysis (https://imagej.net/plugins/directionality) was applied to the filtered image (ImageJ→ Analyze → Directionality). The plugin parameters were set for Fourier component analysis and the number of bins was set to 90. The plugin computes a histogram indicating the amount of structures in a given direction. Images with completely isotropic content are expected to give a flat histogram, whereas images in which there is a preferred orientation are expected to give a histogram with a peak at that orientation (**Supplementary Figure S3B**). Numerical values of the angular dispersion were graphically plotted (**Supplementary Figure S3C**).

**Gray level co-occurrence matrix (GLCM)**

Gray level co-occurrence matrix (GLCM) textures were calculated using FIJI and a custom macro that expedited and automated the analysis. The custom macro utilized the Texture Analyzer plugin (Julio E. Cabrera, version v0.4 2006/07/07) to calculate following texture parameters: (a) Angular Second Moment (ASM) and Energy measures the number of repeated pairs. The energy (square root of ASM) will be low if the occurrence of repeated pixel pairs is low. (b) Contrast is a measure of the local contrast of an image and will be low if the gray levels of each pixel pair are similar. (c) Correlation measures the linear dependency of gray levels on those of neighboring pixels. (d) Inverse Difference Moment (IDM) is related to the smoothness or homogeneity across the image and will be low if the gray levels of the pixel pairs are not similar. (e) Entropy measures the randomness of a gray level distribution and will be high if the gray levels are distributed randomly throughout the image (Rentchler et al. 2019, Reznikov et al. 2013). For each region of interest imaged, the five GLCM textures were calculated for forward image taken from each stack. Additionally, the images were rotated 45°, 90° and 135° and the GLCM textures were then re-calculated. The GLCM values from the horizontal (0°), vertical (90°) and diagonal (45°, 135°) measurements were then averaged.

**Statistics**

The statistical significance was calculated with ordinary one-way ANOVA, P<0.05 considered as statistically significant. Sample size was 5-7 slides per genotype.

**Supplementary Figures**

**
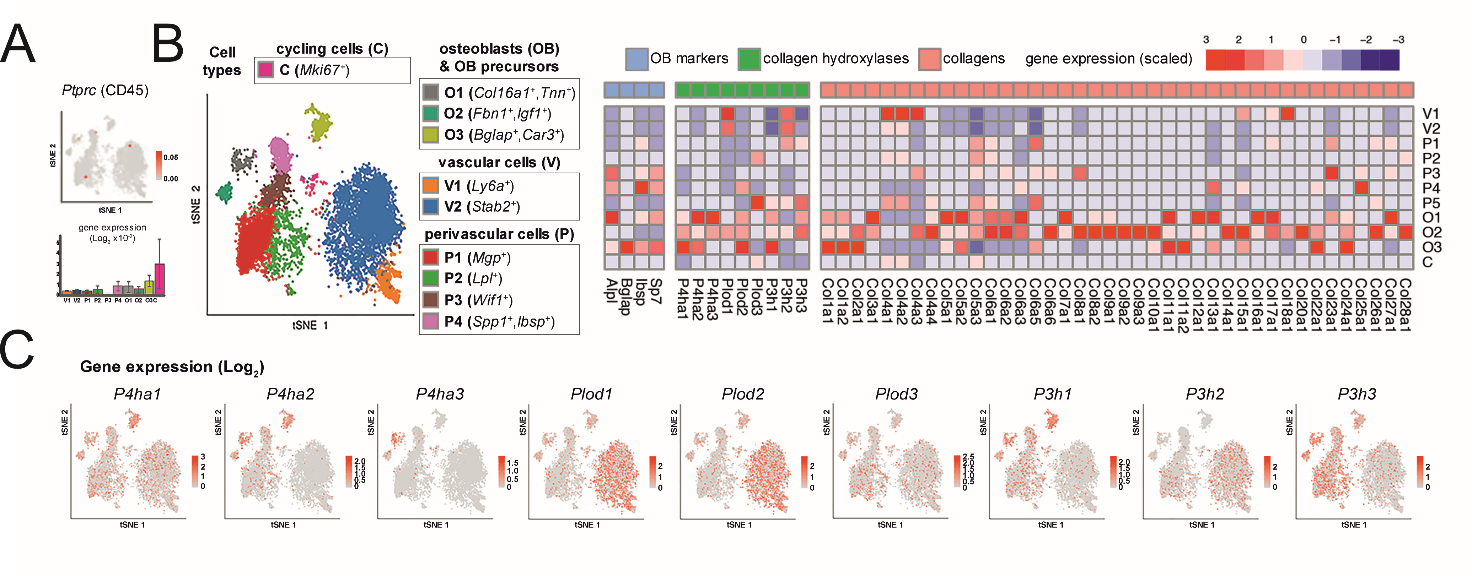
**

**Supplementary Figure S1. Expression of collagen P4H α subunits, other collagen hydroxylases and different collagen types in the mouse bone marrow at single-cell resolution.** (A-B) Single cell RNAseq reveals three osteoblast (OB) clusters marking the process of differentiation from osteogenic precursors (O2) to mature OB (O3) and spanning the myeloid-supportive population of O1 which resembles myofibroblasts of myeloid origin. Other clusters include vascular (V1-V2) and perivascular (P1-P4) lineages with heterogeneous expression of collagen hydroxylases and collagens. Expression of the C-P4H α subunits varies considerably along the OB trajectory with *P4ha1* and *P4ha2* counts increasing in tune with OB commitment while *P4ha3* marks almost exclusively the O1 population. As expected, collagen expression is distributed between mature (O3) OBs (*Col1a1*, *Col1a2* and *Col2a1*) and vascular cells (the basal lamina marker collagen IV, *Col4a1*, *Col4a2* and *Col4a3*). (C) Expression of the different collagen hydroxylases varies across the different stromal cell types, ranging from ubiquitous (*P4ha1* and *Plod3*) to cell-specific such as *P4ha3* (OBs), *Plod2* (vascular cells) or *P3h3* (perivascular cells and OBs).


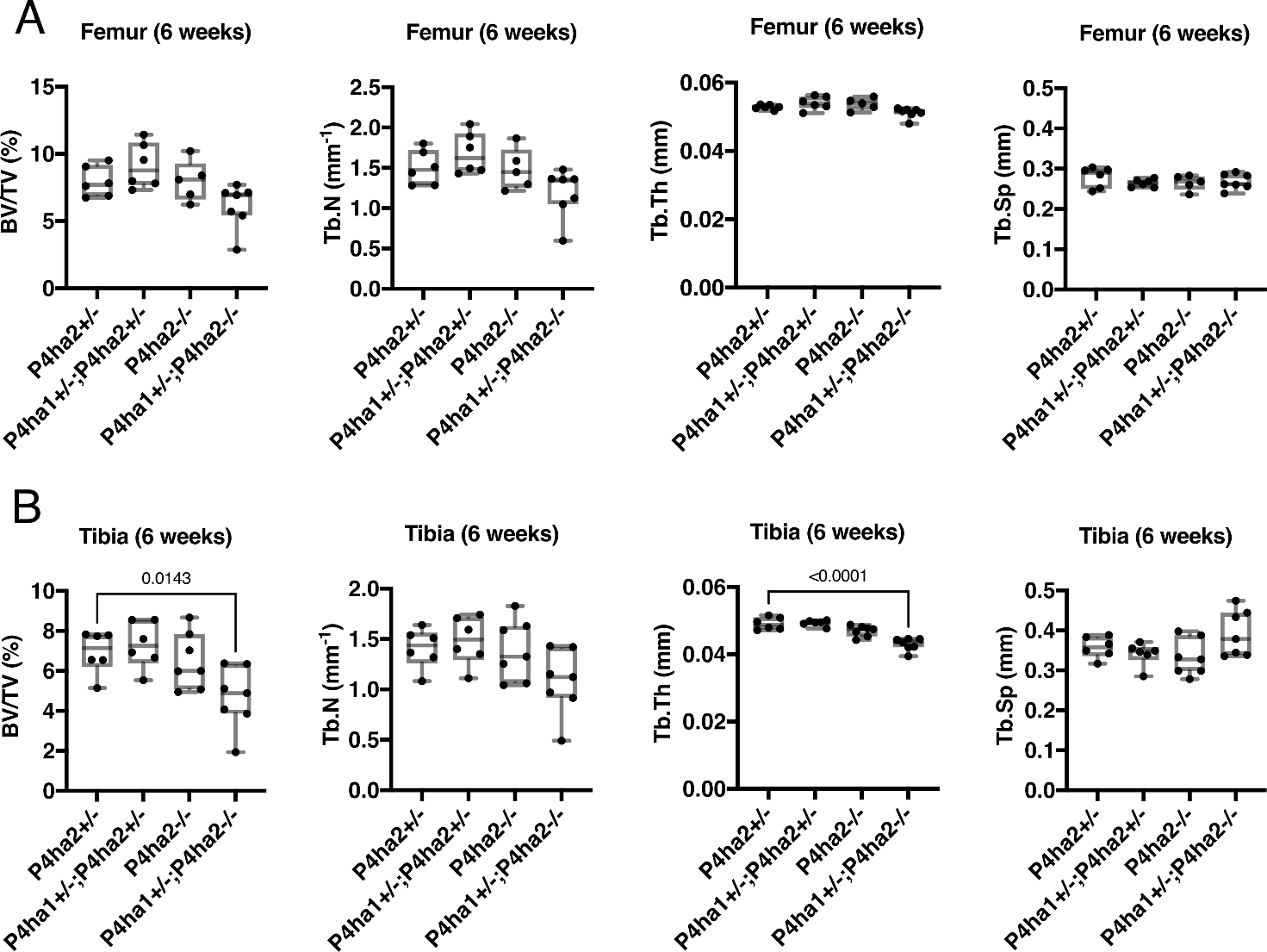


**Supplementary Figure S2. Decreased trabecular bone volume in the tibiae but not femur of 6-week-old *P4ha1*^+/-^;*P4ha2*^-/-^ mice.** (A-B) Quantification of bone volume fraction (BV/TV), trabecular number (Tb.N), trabecular thickness (Tb.Th) and trabecular separation (Tb.Sp) in the distal femur (A) and the proximal tibia (B) at six weeks of age. The data are shown as box and whisker plots including all individual data points, median and interquartile range (25^th^ to 75^th^ percentile). Statistical analysis was done with one-way ANOVA followed by post hoc Dunnett’s multiple comparisons test against the control *P4ha2*^+/-^ mice, n = 5-7 mice/genotype. Statistically significant p-values are shown in the graphs.

*
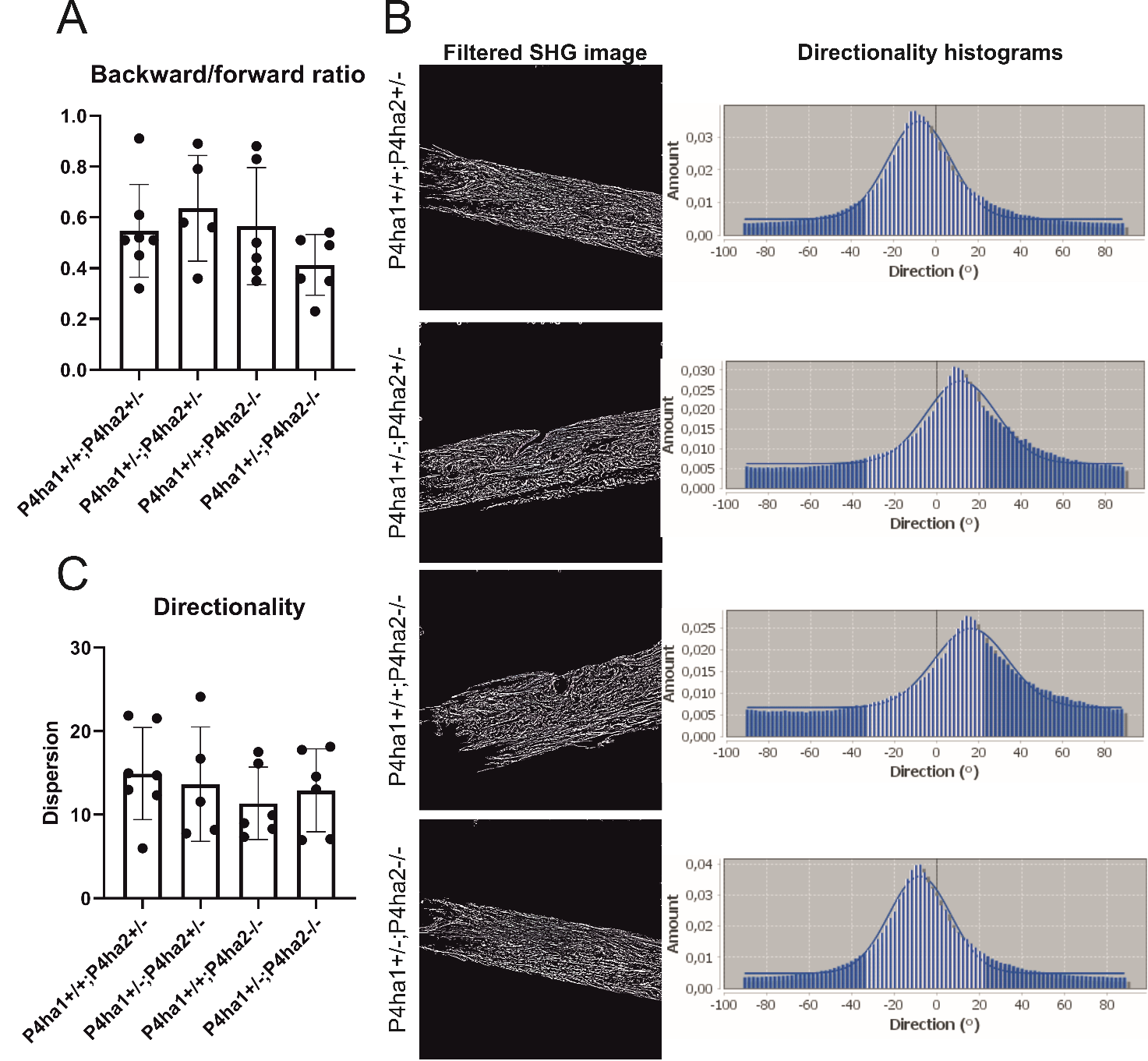
*

**Supplementary Figure S3. Backward/Forward ratio of SHG images.** (A) No significant changes in the type I/III collagen ratio are observed suggesting that C-P4H deficiency does not affect the collagen fibril composition in the tibiae. (B) Representative forward direction images and the Gaussian curves of the Directionality analysis. No phenotypical differences were observed. (C) Numerical values of the dispersion of the Directionality analysis. Dispersion informs how much the direction varies. No significant differences were observed across the genotypes. The data are shown as bar plots including all individual data points. Statistical analysis was done with one-way ANOVA followed by post hoc Dunnett’s multiple comparisons test against the control *P4ha2*^+/-^ mice, n = 5-7 mice/genotype.


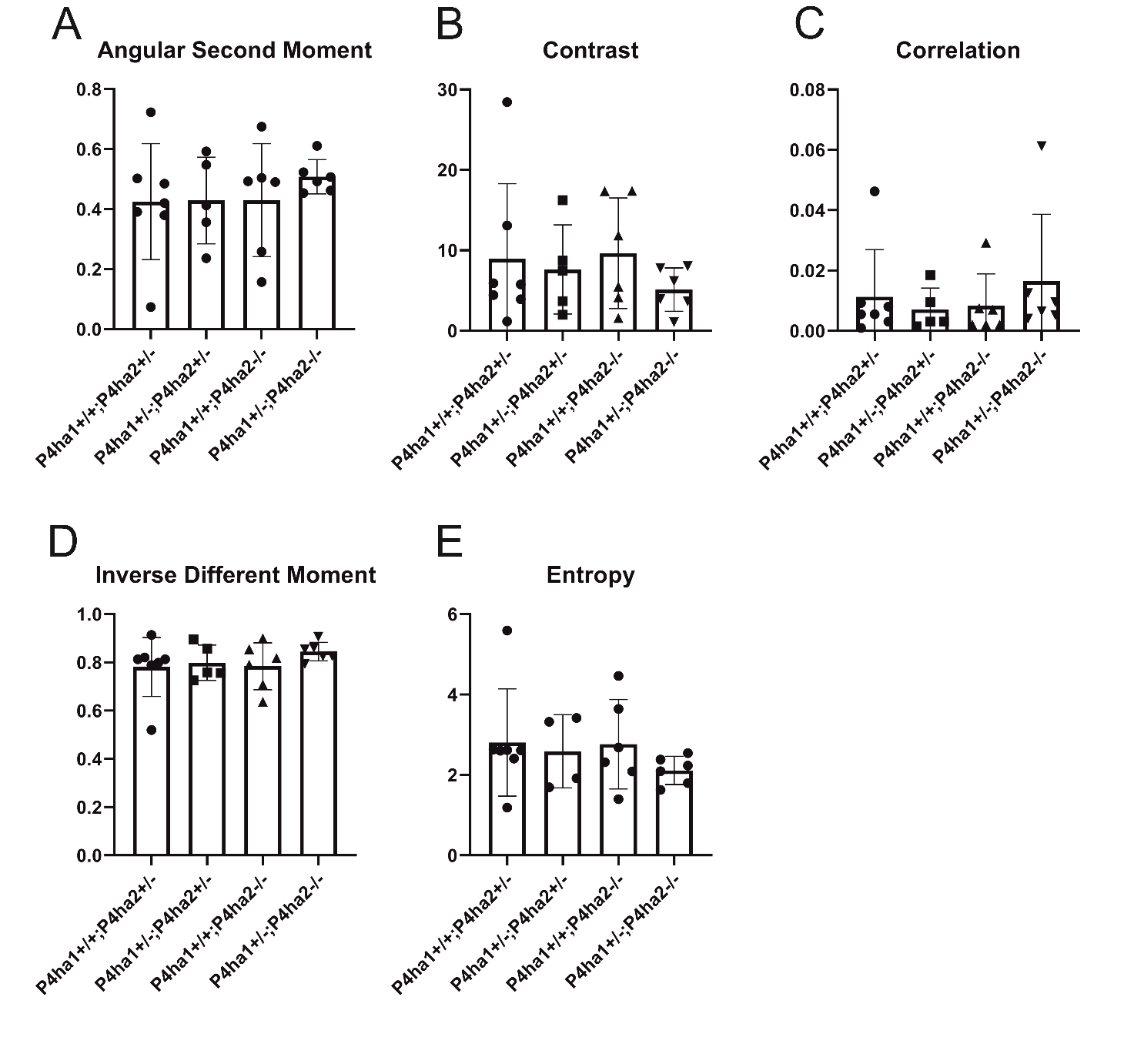


**Supplementary Figure S4. Bar plots of the mean value for each genotype in all five of the textures calculated in the GLCM analysis.** (A) Angular second moment, (B) Contrast, (C) Correlation, (D) Inverse different moment, and (E) Entropy. The data are shown as bar plots including all individual data points. Statistical analysis was done with one-way ANOVA followed by post hoc Dunnett’s multiple comparisons test against the control *P4ha2*^+/-^ mice, n = 5-7 mice/genotype.


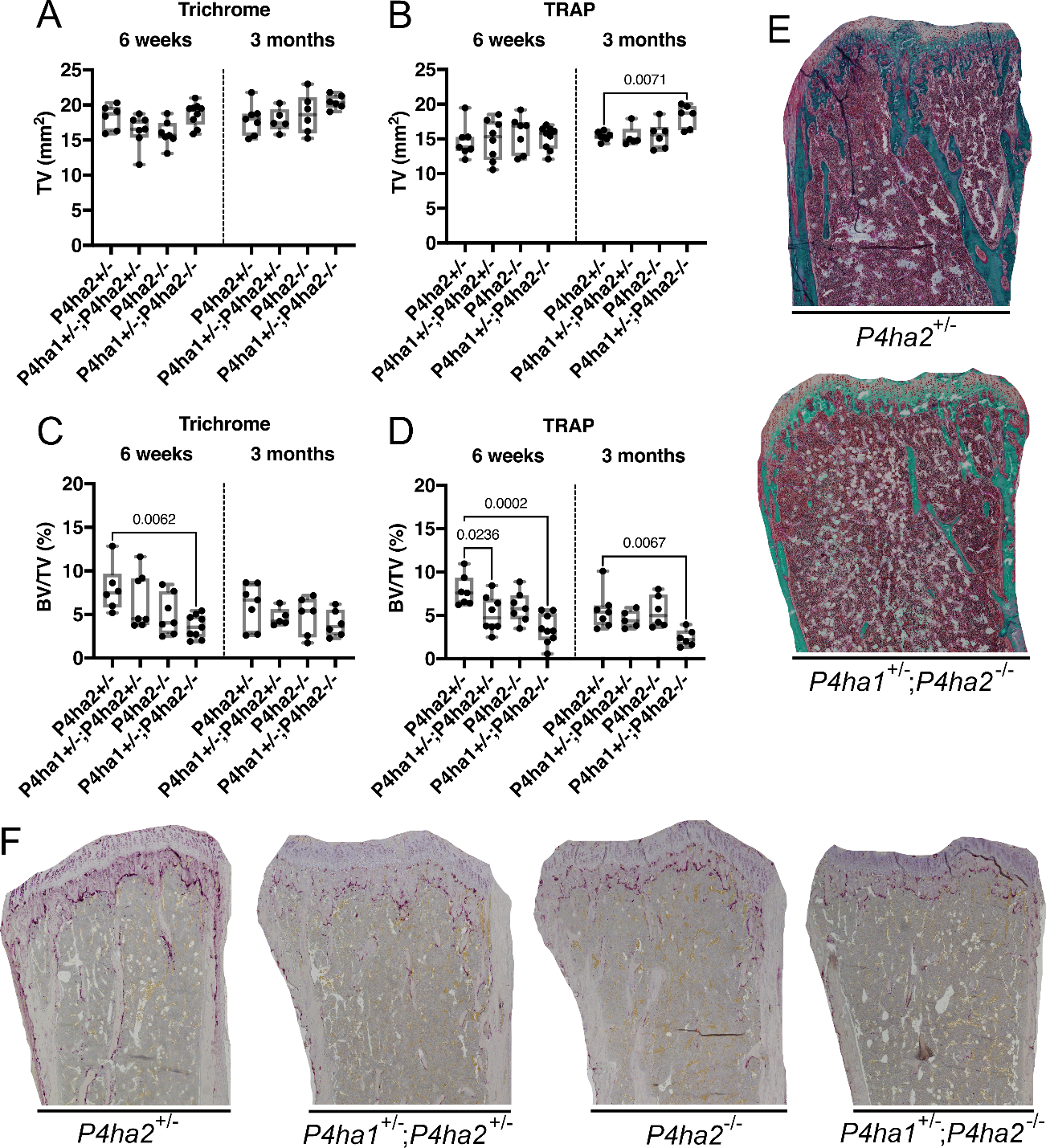


**Supplementary Figure S5. Static histomorphometric measurements of Masson-Goldner’s trichrome and TRAP-stained proximal tibiae at six weeks and three months of age.** (A-B) Tissue volume (TV) in the trichrome (A) and TRAP-stained (B) proximal tibia. (C-D) Bone volume/tissue volume (BV/TV) in the trichrome (C) and TRAP-stained (D) proximal tibia. (E) Representative Masson-Goldner’s trichrome-stained images for the control *P4ha2^+/-^* mice and *P4ha1*^+/-^;*P4ha2*^-/-^ mice at 6 weeks of age. (F) Representative TRAP-stained images for each genotype at 6 weeks of age. The data are shown as box and whisker plots including all individual data points, median and interquartile range (25^th^ to 75^th^ percentile). Statistical analysis was done with one-way ANOVA followed by post hoc Dunnett’s multiple comparisons test against the control *P4ha2*^+/-^ mice, n = 5-9 mice/genotype. Statistically significant p-values are shown in the graphs.


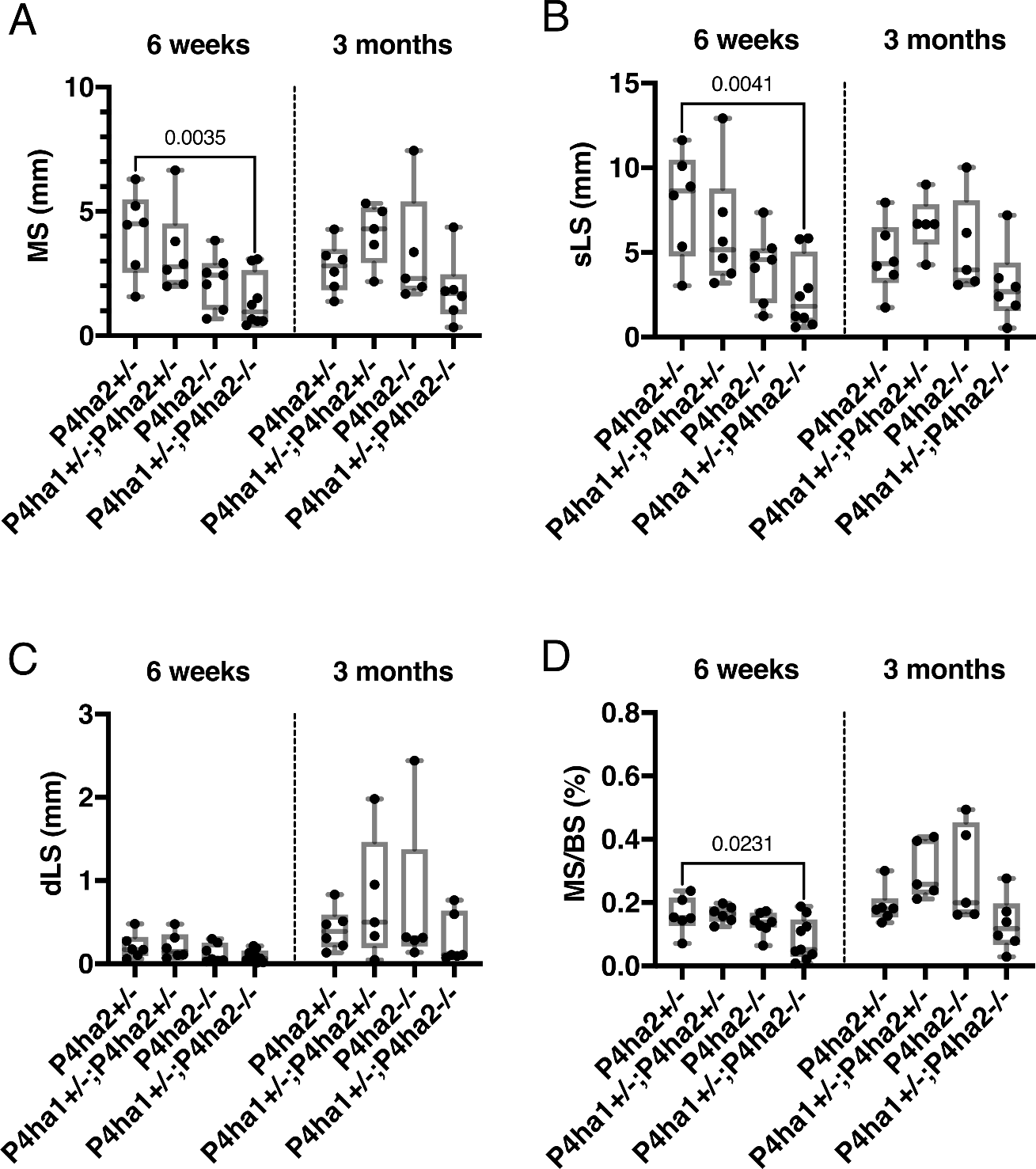


**Supplementary Figure S6. Reduced mineralized surface in the *P4ha1*^+/-^;*P4ha2*^-/-^ tibiae at six weeks but not at three months of age.** To perform dynamic histomorphometric analyses, the mice were injected i.p. with 40 mg/kg calcein at six and two days prior to sacrifice. (A-D) Quantification of mineral surface (MS) (A), single-labelled surface (sLS) (B), double-labelled surface (dLS) (C), and mineralizing surface/bone surface (MS/BS) (D) in the proximal tibia at six weeks and three months of age. The data are shown as box and whisker plots including all individual data points, median and interquartile range (25^th^ to 75^th^ percentile). Statistical analysis was done with one-way ANOVA followed by post hoc Dunnett’s multiple comparisons test against the control *P4ha2*^+/-^ mice, n = 5-9 mice/genotype. Statistically significant p-values are shown in the graphs.


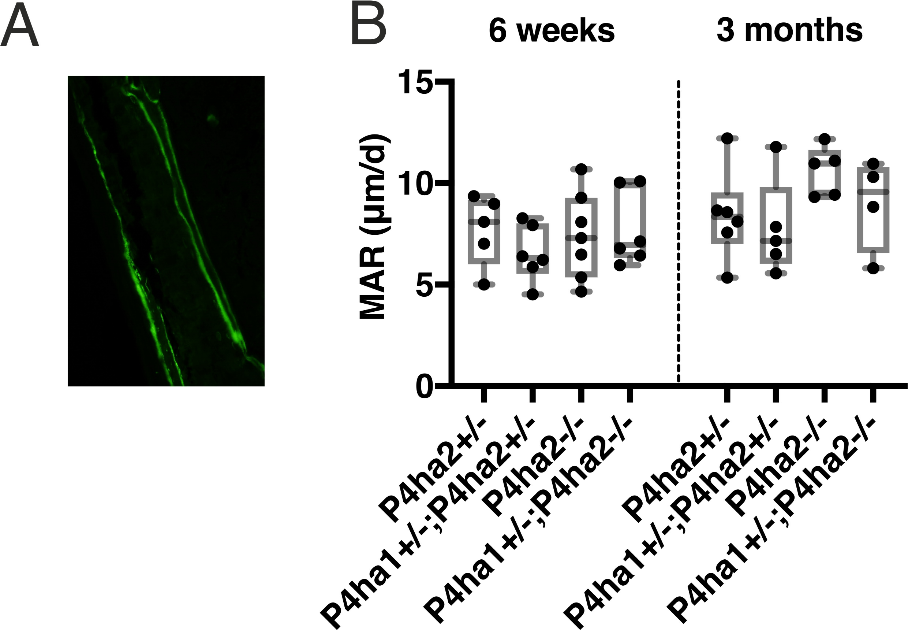


**Supplementary Figure S7. Mineral apposition rate (MAR) at the periosteal and endosteal surfaces.** (A) Cortical calcein fluorescence signal at tibial midshaft showing both periosteal and endosteal surfaces at six weeks of age. (B) Cortical MAR at six weeks and three months of age. The data are shown as box and whisker plots including all individual data points, median and interquartile range (25^th^ to 75^th^ percentile). Statistical analysis was done with one-way ANOVA followed by post hoc Dunnett’s multiple comparisons test against the control *P4ha2*^+/-^ mice, n = 4-7 mice/genotype.


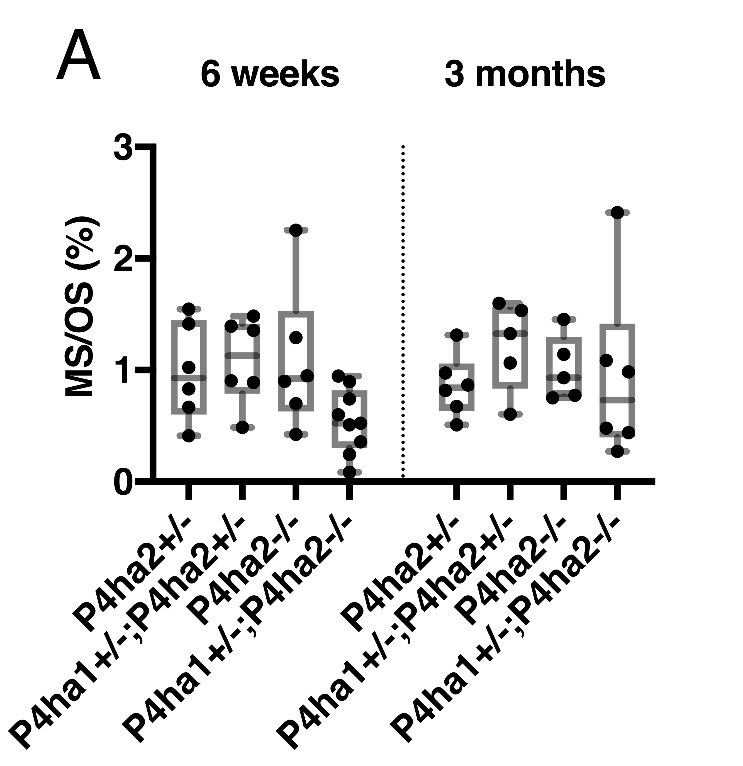


**Supplementary Figure S8. Analysis of the fraction of osteoid surface undergoing mineralization.** (A) Mineralizing surface/osteoid surface (MS/OS) in the proximal tibia at six weeks and three months of age. The data are shown as box and whisker plots including all individual data points, median and interquartile range (25^th^ to 75^th^ percentile). Statistical analysis was done with one-way ANOVA followed by post hoc Dunnett’s multiple comparisons test against the control *P4ha2*^+/-^ mice, n = 5-9 mice/genotype.


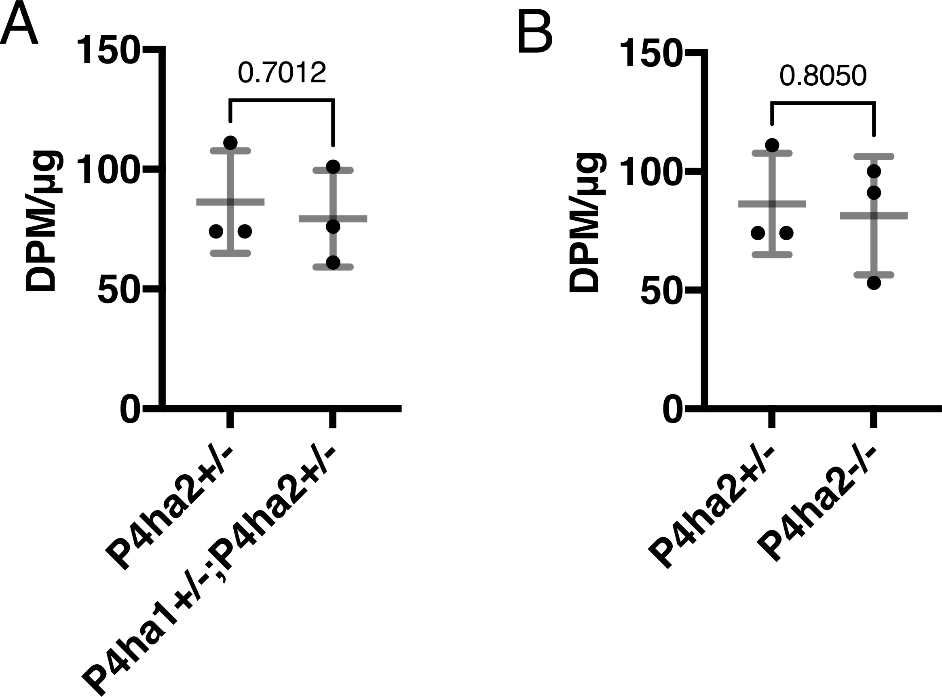


**Supplementary Figure S9. Total C-P4H activity in the double heterozygous *P4ha1*^+/-^;*P4ha2*^+/-^ and the C-P4H-II knockout *P4ha2*^-/-^ osteoblasts.** (A-B) Osteoblasts were isolated from both tibiae and femurs at five weeks of age to measure the total C-P4H activity using [^14^C]proline-labeled non-hydroxylated procollagen α chains of chick type I collagen as substrate. Total C-P4H activity in *P4ha1*^+/-^;*P4ha2*^+/-^ (A) and *P4ha2*^-/-^ (B) osteoblasts. The data are shown as box and whisker plots including all individual data points, median and interquartile range (25^th^ to 75^th^ percentile). Statistical analysis was done with Student’s t test, n = 3 mice/genotype. The p-values are shown in the graphs.

**Supplementary References**

LaComb R, Nadiarnykh O, Townsend SS & Campagnola PJ. (2008) Phase Matching considerations in Second Harmonic Generation from tissues: Effects on emission directionality, conversion efficiency and observed morphology. Opt Commun 281(7): 1823-1832.

Rentchler EC, Gant KL, Drapkin R, Patankar M & J. Campagnola P. (2019) Imaging Collagen Alterations in STICs and High Grade Ovarian Cancers in the Fallopian Tubes by Second Harmonic Generation Microscopy. Cancers (Basel) 11(11).

Reznikov N, Almany-Magal R, Shahar R & Weiner S. (2013) Three-dimensional imaging of collagen fibril organization in rat circumferential lamellar bone using a dual beam electron microscope reveals ordered and disordered sub-lamellar structures. Bone 52(2): 676-683.

Tilbury K, Lien C, Chen S & Campagnola P. (2014) Differentiation of Col I and Col III Isoforms in Stromal Models of Ovarian Cancer by Analysis of Second Harmonic Generation Polarization and Emission Directionality. Biophysical Journal 106(2): 354-365.
